# A Comprehensive Review of the Ethnotraditional Uses and Biological and Pharmacological Potential of the Genus *Mimosa*

**DOI:** 10.3390/ijms22147463

**Published:** 2021-07-12

**Authors:** Ismat Majeed, Komal Rizwan, Ambreen Ashar, Tahir Rasheed, Ryszard Amarowicz, Humaira Kausar, Muhammad Zia-Ul-Haq, Luigi Geo Marceanu

**Affiliations:** 1 Department of Chemistry, Government College Women University, Faisalabad 38000, Pakistan; ismatmajeed123@gmail.com (I.M.); ambreenashar2013@gmail.com (A.A.); 2 Department of Chemistry, University of Sahiwal, Sahiwal 57000, Pakistan; komal.rizwan45@yahoo.com; 3 School of Chemistry and Chemical Engineering, Shanghai Jiao Tong University, Shanghai 200240, China; masil@sjtu.edu.cn; 4 Department of Chemical and Physical Properties of Food, Institute of Animal Reproduction and Food Research, Polish Academy of Sciences, Tuwima Street 10, 10-748 Olsztyn, Poland; 5 Department of Chemistry, Lahore College for Women University, Lahore 54000, Pakistan; humairakausar1@gmail.com; 6 Office of Research, Innovation & Commercialization, Lahore College for Women University, Lahore 54000, Pakistan; ahirzia@gmail.com; 7 Faculty of Medicine, Transilvania University of Brasov, 500019 Brasov, Romania; marceanu@gmail.com

**Keywords:** *Mimosa*, genus, Fabaceae, pharmacology, nutrition, leaves

## Abstract

The *Mimosa* genus belongs to the Fabaceae family of legumes and consists of about 400 species distributed all over the world. The growth forms of plants belonging to the Mimosa genus range from herbs to trees. Several species of this genus play important roles in folk medicine. In this review, we aimed to present the current knowledge of the ethnogeographical distribution, ethnotraditional uses, nutritional values, pharmaceutical potential, and toxicity of the genus *Mimosa* to facilitate the exploitation of its therapeutic potential for the treatment of human ailments. The present paper consists of a systematic overview of the scientific literature relating to the genus *Mimosa* published between 1931 and 2020, which was achieved by consulting various databases (Science Direct, Francis and Taylor, Scopus, Google Scholar, PubMed, SciELO, Web of Science, SciFinder, Wiley, Springer, Google, The Plant Database). More than 160 research articles were included in this review regarding the *Mimosa* genus. *Mimosa* species are nutritionally very important and several species are used as feed for different varieties of chickens. Studies regarding their biological potential have shown that species of the *Mimosa* genus have promising pharmacological properties, including antimicrobial, antioxidant, anticancer, antidiabetic, wound-healing, hypolipidemic, anti-inflammatory, hepatoprotective, antinociceptive, antiepileptic, neuropharmacological, toxicological, antiallergic, antihyperurisemic, larvicidal, antiparasitic, molluscicidal, antimutagenic, genotoxic, teratogenic, antispasmolytic, antiviral, and antivenom activities. The findings regarding the genus *Mimosa* suggest that this genus could be the future of the medicinal industry for the treatment of various diseases, although in the future more research should be carried out to explore its ethnopharmacological, toxicological, and nutritional attributes.

## 1. Introduction

**Figure 1:**
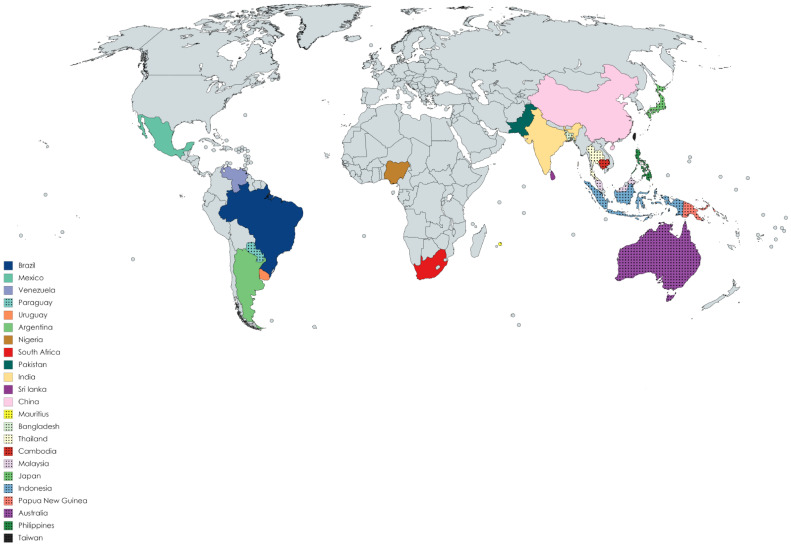
Ethnogeographical distribution of *Mimosa* species.

The *Mimosa* genus belongs to the *Fabaceae* family of legumes (subfamily: *Mimosoideae*) and consists of almost 400 species of shrubs and herbs [1]. The species are distributed mainly in Bangladesh, Indonesia, Malaysia, Japan, India, Pakistan, Sri Lanka, China, Cambodia, Taiwan, Africa (Nigeria, Mauritius, and Reunion Island), Australia, Brazil, Venezuela, Mexico, Philippines, Cuba, northern Central America, Paraguay, Argentina, Uruguay, Thailand, several Pacific Islands, Papua New Guinea, and North America [2,3,4,5,6,7,8,9,10,11]. Figure 1 presents the ethnogeographical distribution of the *Mimosa* species in different countries of the world. Almost 20–25 species of this genus are well known to the world, including *Mimosa tenuiflora* (Wild.) pior, *Mimosa pudica* L., *Mimosa pigra* L., *Mimosa caesalipiniifolia* Benth., *Mimosa hamata* Willd., *Mimosa diplotricha* Sauvalle, *Mimosaa xanthocentra* Mart., *Mimosa artemisiana* Heringer and Paula, *Mimosa invisa* Mart. ex Colla, *Mimosa scabrella* Benth., *Mimosa somnians* Humb. and Bonpl. ex Willd., *Mimosa bimucronata* (DC.) Kuntze, *Mimosa verrucosa* Benth., *Mimosa arenosa* (Willd.) Poir., *Mimosa humilis* Willd., *Mimosa rubicaulis* Lam., *Mimosa linguis*, and *M. albida* Willd. *Mimosa ophthalmocentra* Mart. ex Benth. (http://mpns.kew.org/MPNS.kew.org 2018; www.theplantlist.org 2017) (Accessed on 18 May 2021). Leaves of this genus may be bipinnate or binate, compound or branched, with one or two pairs of branchlets or much larger branched leaves. Some species have the ability to fold their leaves when touched, with *M. pudica* being one common example. The flowers may be pink and globular in the form of clusters and with prickles or may be white and grouped in dense heads 3–6.5 mm long. The fruit are lance-shaped with 2–6 articulations. The fruit wall is compressed between the seeds. Huge amounts of starch and calcium oxalate crystals are present in the bark [12]. Some species are prickly leguminous shrubs [13]. The plants of this genus usually grow across roadsides, walkways, marshes, and hillsides, and on margins of rivers and lakes on wet soil, where several individuals can form dense aggregations [14]. The plants are commonly used for ornamental purposes. They also serve as sleeping shelters for animals [15]. Economically, their wood is used for fence posts, firewood, coal, plywood, particle board, and lightweight containers, and more recently has been introduced in furniture and flooring [16]. Leaves of the plants are used for poultry diets [17]. In the food industry, the leaves are used as additives, while in the leather and textile industry they are used as colorants [18,19]. The plants provide wood for market purposes and add nitrogen in warm-climate silvopasture systems [20]. This genus has remarkable economic importance in the cosmetics industry [21]. Traditionally, species of this genus are used in folklore medicines for the treatment of various ailments, including head colds, wounds, toothaches, jaundice, eye problems, fever, weak heart, skin burns, asthma, diarrhea, piles, gastrointestinal ailments, liver disorders (such as hepatitis and diuresis), and respiratory [22,23,24,25,26] disorders. They are also used as coagulants and in tonics for urinary complaints. The genus *Mimosa* has been demonstrated to possess various pharmacological activities, including antiseptic, antimicrobial [27,28], antioxidant [29,30,31,32,33,34], anticonvulsant [35], antifertility [36], antigout [37], anti-inflammatory [38,39,40], antinociceptive [41,42], antiulcer [43], antimalarial [44], antiparasitic [45], antidiabetic [46,47], anticancer [48,49], antidepressant [50], antidiarrheal [51,52], antihistamic [53], wound-healing [54], antispasmolytic [55], hypolipidemic [56], hepatoprotective [57], hypolipidiemic [58], antivenom [40], antiproliferative [59], antiviral [60], and aphrodisiac activities [61]. Phytochemicals studies of plants have revealed the presence of secondary metabolites, such as alkaloids, tannins, flavonoids, terpenoids, saponins, steroids [62,63], and coumarins [64]. The green synthesis of nanoparticles is cheap, simple, comparatively and reproducible, resulting in the production of more stable and useful materials [65]. The *Mimosa* genus has been used in the green synthesis of pharmacologically important gold [66], silver [67], iron [68], cadmium [69], platinum [70], and zinc oxide [71]-based nanoparticles; however, data regarding all species of the *Mimosa* genus have not been compared and organized to date in proper review form (to the best knowledge of the authors). Our research group is currently working on compiling data regarding bioactive constituents isolated from the genus *Mimosa* and their pharmacological effectiveness.

## 2. Materials and Methods

A detailed bibliographic study that included papers published from 1931 to 2020 was carried out. Several databases (Science Direct, Francis and Taylor, Scopus, Google Scholar, PubMed, SciELO, Web of Science, SciFinder, Wiley, Springer, Google, and The Plant Database) were explored in order to collect information on this genus. Various books, full text manuscripts, and abstracts were consulted. The genus name and the synonyms and scientific names of *Mimosa* species were used as keywords. The scientific names of all plants of the genus Mimosa and their synonyms were validated using a standard database (http://mpns.kew.org/MPNS.kew.org 2018; www.theplantlist.org 2017) (accessed on 15 May 2021).

## 3. Nutritional Potential of Genus *Mimosa*

**Figure 2:**
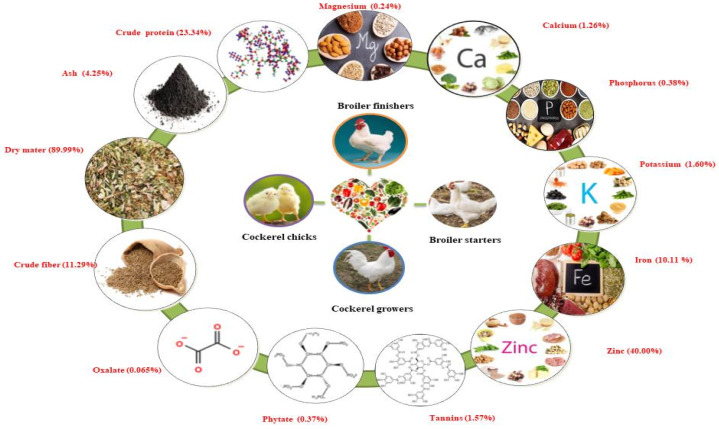
Schematic presentation of the nutritional value of the *Mimosa* genus. Note: % represents percentage of dry matter content.

Nworgu and Egbunike [17] reported on the nutritional potential of *M. invisa* leaves by preparing meals for different varieties of cockerel chicks, cockerel growers, broiler starters, and finishers within the years 2004–2009. The diets were formulated for cockerel chicks, broiler starters, and finishers. Leaves were found to be rich in crude protein (23.34%), ash (4.25%), dry matter (89.99%), crude fiber (11.29%), nitrogen-free extract (58.74%), oxalate (0.065%), phytate (0.37%), and tannins (1.57%). High mineral elements were found in *M. invisa* leaves, including zinc (40.00% of DM), iron (10.11% of DM), potassium (1.60% of DM), calcium (1.26% of DM), phosphorus (0.38% of DM), and magnesium (0.24% of DM). Inclusion of more than 20 g/kg *M. invisa* leaf meal to the diets of cockerel chicks and cockerel growers resulted in decreased feed intake, weight gain, and feed conversion ratio. Dietary inclusion of leaf meal in broiler starters and finishers resulted in significant reductions in feed, weight gain, and feed conversion ratio. These results were found to be progressive and comparable within different chicken types. A schematic illustration of the nutritional value of the Mimosa genus is presented in Figure 2.

Rajan et al. [72] measured trace elements present in *M. pudica* leaves with the help of the proton-induced X-ray emission (PIXE) technique. PIXE analysis revealed that trace elements of Fe = 308.467 mg/L, Mn = 65.664 mg/L, Zn =18.209 mg/L, Cu = 10.707 mg/L, Co = 2.025 mg/L, and V= 0.059 mg/L were present in *M. pudica* leaves. These trace elements are used for curing skin diseases, especially infections on the legs and between the fingers, and are also taken orally. Yongpisanphop et al. [73] estimated lead contamination levels in *M. pudica* roots. The lead contamination level found to be in the root sample was 826 mg/kg, as compared to 496 mg/kg in the soil sample. There are no reports available regarding the trace elements and mineral compositions of other *Mimosa* species.

## 4. Ethno-Traditional Uses of Genus *Mimosa*

**Figure 3:**
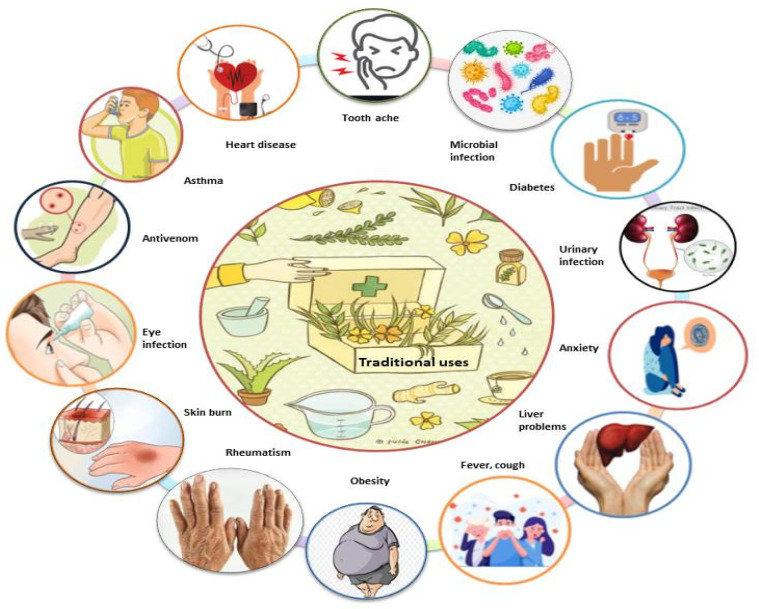
Schematic illustration of the traditional uses of the genus *Mimosa*.

Various species of Mimosa, including *M. tenuiflora*, *M. pudica*, *M. pigra*, *M. caesalpiniifolia*, *M. hamata*, *M. rubicaulis*, *M. somnians*, *M. bimucronata*, *M. linguis*, *M. humilis*, *M. invisa*, *M. arenosa*, *M. ophthalmocentra*, *M. verrucosa*, and *M. albida*, have been reported to be used in traditional medicine for the treatment of various ailments (Figure 3). Due to their potential benefits in phytomedicines, all parts of this genus are used in traditional systems of medicine in Mexico, Brazil, India, Bangladesh, China, Indonesia, Madagascar, South America, and tropical Africa for countless ailments, including toothaches, head colds, and eye problems. M. tenuiflora is a perennial shrub or tree commonly known as skin tree or Jurema-preta in many parts of the world. In Northeastern Brazil, the bark of M. tenuiflora is used in a religious drink called Yurema [74,75], while the leaves, stem, and flowers are used to relieve fever, menstrual colic, headache, hypertension, bronchitis, and coughs [41,55,76,77,78,79,80,81]. In Mexico, its stem bark is used to treat skin burns, lesions, and inflammation [24]. 

*M. pudica* is a creeping perennial or annual flowering plant and is the most famous plant of this genus; it is commonly known as touch-me-not, sensitive plant, or shy plant. In Mexico, M. pudica is used for the treatment of depression, anxiety, premenstrual syndrome, menorrhagia, skin wounds, diarrhea, and rheumatoid arthritis [50,82,83,84,85]. Ayurveda and Unani are the two main medical systems in India, in which *M. pudica* leaves and roots are used for prevention of vaginal and uterine infections [86], ulcers, bile, leprosy, fever, small pox, jaundice, and piles [87,88]. The seeds are combined with sugar and used to control skin and venereal diseases [72]. Indians use it for the removal of kidney stone (vesicle calculi) myalgia, rheumatism, uterus tumors, and odema-type disorders [89]. The leaves are commonly used in Bangladesh as one of the ingredients to control piles, diarrhea, persistent dysentery, and convulsion of children [90]; additionally, the root extracts have antivenomic properties [91]. In China, women use its herbal paste in the form of a solution to narrow their vaginas [92], and it is also used as a dental powder to treat gingiva and bad breath [93]. *M. pigra* is a leguminous shrub known as giant sensitive tree and bashful plant. The people of Africa, the America, Indonesia, and Mexico use *M. pigra* to deal with several health disorders, such as liver ailments, hepatitis and respiratory disorders [94], and snakebite [95]. It is also used for mouthwash to treat toothaches and in eye medicines [96]. Additionally, the leaves are used by the local people of Bangladesh to lower their blood sugar [97], while Indonesian people use the roasted ground leaves to stabilize a weak heart or weak pulse [98,99].

*M. caesalpiniifolia* is a spiny, deciduous tree or shrub with white flowers that is commonly found in Brazil. Its bark and flowers are used as an effective remedy to prevent skin infections, injuries [100], and hypertension [77].

*M. hamata* is a flowering shrub found in India that is known as Jinjani, which is used as animal feed. The roots and leaves are used in customary medications for the treatment of numerous health ailments, such as jaundice, diarrhea, coagulant, fever, wounds, piles, gastrointestinal and liver disorders (such as hepatitis), and respiratory issues [3], as well as being used in tonics for urinary complaints. A paste made from the leaves is applied to reduce glandular swelling, piles, sinus issues, and sores [101,102]. The seed extract is used as a blood purifier [33,103].

*M. albida* is a leguminous shrub commonly found in rain forests. In Brazil, roots are used for cardiovascular and renal system disorders [104], as well as inflammation of the uterus [105]. In Mexico, the leaves are used for treatment of chronic pain [106]. In Honduras, women use its roots in abortifacient agents [107]. Different species of *Mimosa* are known by different names in different countries. Ethnobotanical information for this genus based on their geographic distribution, along with the plant parts and their corresponding medicinal properties, are listed in Table 1.

## 5. Pharmacological Activities of Genus *Mimosa*

During the past decades, the genus *Mimosa* has been extensively studied for its broad biological and pharmacological potential. Different preparations and extracts from this genus have demonstrated multiple health benefits and pharmacological effects, including antimicrobial, antioxidant, anticancer, wound-healing, hypolipidemic, anti-inflammatory, hepatoprotective, antinociceptive, antiepileptic, neuropharmacological, toxicological, antiallergic, antihyperurisemic, larvicidal, antiparasitic, molluscicidal, antimutagenic, genotoxic–teratogenic, antispasmolytic, antiviral, and antivenom activities. These pharmacological effects have been studied through in vitro and in vivo assays. These activities are presented in detail in this review article (Table 2).

### 5.1. Antimicrobial Activity

Valencia-Gómez et al. [129] determined the antibacterial activity of biofilms made from chitosan and *M. tenuiflora* bark. Composite biofilms in different concentrations (100:0, 90:10, 80:20, and 70:30) successfully inhibited the growth of *E. coli* and *M. lysodeikticus*. Souza-Araújo et al. [130] measured the antimicrobial activity of pyroligneous acid (PA) obtained from slow pyrolysis of wood of *M. tenuiflora* against *E. coli*, *P. aeruginosa*, *S. aureus*, *C. albicans,* and *C. neoformans* using the agar diffusion method. The growth of all microorganisms was inhibited by pyroligneous acid at different tested concentrations (20, 50, and 100%), whereas gentamicin was used as a standard drug. The antimicrobial potential of EtOH (95%) extract of *M. tenuiflora* bark against different bacterial and fungal strains has been reported. Active doses of the extract inhibited the growth of *E. coli*, *B. subtilis*, *M. luteus,* and *P. oxalicum***.** Gonçalves et al. [131] reported the antimicrobial potential of the hydroalcoholic extract of *M. tenuiflora* bark against various bacterial strains using the agar well diffusion method. The results revealed that the extract successfully inhibited the growth of *S. pyogenes*, *P. Mirabilis*, *S. sonnei*, *S. pyogenes,* and *Staphylococcus* spp. Padilha et al. [132] described the antibacterial activity of the EtOH extract of *M. tenuiflora* stem bark against *S. aureus* by using the minimum inhibitory concentration (MIC) with the agar dilution method and time-kill assay. At concentrations up to 4x MIC, only a bacteriostatic effect was observed, while at 8.x MIC a fast bactericidal effect was observed [133]. The minimum inhibitory concentration shown by active doses of 95% *M. tenuiflora* EtOH extract against *S. epidermidis* and *A. calcoaceticus* were >10.0 μg/mL, *S. aureus* and *M. luteus* = 10.0 μg/mL, *E. coli* and *K. pneumonia* = 20.0 μg/mL, and *C. albicans* = 70.0 μg/mL [134]. The antimicrobial potential of BuOH, MeOH, and EtOAc extracts of *M. tenuiflora* bark against *S. aureus*, *E. coli,* and *C. albicans* has been reported [135]. De Morais-Leite et al. [136] determined the antibacterial potential of the EtOH extract of *M. tenuiflora* bark via the minimum inhibitory concentration (MIC) and the minimum bactericidal concentration (MBC) values against *S. aureus* (ATCC 25.925 and ATCC 25.213), *E. coli* (ATCC 8859 and ATCC 2536), and *P. aeruginosa* (ATCC 25.619). *S. aureus* (ATCC 25.925) and *P. aeruginosa* (ATCC 25.619) showed MIC and MBC values of 128 and 256 μg/mL, respectively, while *S. aureus* (ATCC 25.213) showed MIC = 512 and MBC = 1024 μg/mL. For *E. coli* (ATCC 8859) and *E. coli* (ATCC 2536), the observed values were MIC = 1024 and MBC ˃1024 μg/mL. Silva and his colleagues reported on the antimicrobial potential of the EtOH extract of *M. tenuiflora* bark using the minimum inhibitory concentration (MIC) values against *S. aureus*, *E. coli*, *C. albicans*, and *T. interdigitale*. Lower MIC values were observed against *S. aureus* [137].

Racadio [116] and Molina [117] reported on the antimicrobial activity of the EtOH extract of *M. pudica* leaves against *S. aureus*, *B. subtilis*, and *C. albicans* using the Kirby–Bauer disc diffusion method. Inhibition zones were observed against *S. aureus* = 21.8 mm (4.61%), *B. subtilis* = 23.7 mm (9.56%), and *C. albicans* = 6.1 mm (1.96%). Nagarajan et al. [139] determined the antibacterial activity of Aq. extracts of *M. pudica* leaves and stems against *E. coli*, *staphylococcus* sp., *Bacillus* sp., *Pseudomonas* sp*.,* and *Streptococci* sp. by disc diffusion method. Zones of inhibition were observed against order *E. coli* (18 mm) *> Bacillus* sp. (12.5 mm) > *Pseudomonas* sp. (12 mm) *> Staphylococcus* sp. (11 mm) > *Streptococcai* sp. (9 mm). Abirami et al. [139] reported on the antimicrobial potential of extracts (ACE, EtOAc, petroleum ether, and Aq.) of *M. pudica* leaves using the well diffusion method. The antimicrobial efficacy levels of all of the extracts were determined against *E. coli*, *P. aeurogiosa*, *L.*, *Bacillus*, *S. typhi*, *S. aureus*, *P. foedians*, *F. oxysporum*, and *P. variotii* at different concentrations of 30, 60, 90, and 120 μL/mL. ACE extract showed a maximum zone of inhibition against *S. aureus,* while Aq. extract showed a maximum activity against *E.* coli. Petroleum ether showed a higher zone of inhibition against *S. typhi.* Durgadevi and Karthika [62] determined the antimicrobial potential of Aq. extract of *M. pudica* leaves by using the agar well diffusion method against *B. cereus*, *E. coli*, *P. valgaris*, *P. auroginosa*, *S. aureus*, *A. flavus*, *A. niger*, *A. terreus*, *Fusarium* sp., and *Penicillium* sp. at different concentrations (25, 50, 75 and 100 mg). The extract showed significant zones of inhibition at 100 mg concentration. Sheeba et al. [140] determined the antibacterial activity of the MeOH extract of *M. pudica* leaves using the disc diffusion method against *P. aeruginosa*, *S. aureus,* and *V. harveyi*. The plant extract showed zones of inhibition against *S. aureus* (10.66 mm), *P. aeruginosa* (8.66 mm), and *V. harveyi* (8.00 mm), while ampicillin was used as the standard antibiotic. Sheeba et al. [140] determined the antimycobacterial activity of MeOH extracts of *M. pudica* leaves against *M. tuberculosis* using disc diffusion and agar well diffusion methods. Extract exhibited a zone of inhibition against *M. tuberculosis* (disc diffusion = 7.00 mm; agar well diffusion method = 4.33 mm). Kakad et al. [141] determined the antibacterial activity of MeOH extract of *M. pudica* leaves against two Gram-positive (*B. subtilis*, *S. aureus)* and three Gram-negative *(P. aeroginosa*, *P. vulgaris*, and *S. typhi)* bacterium using the agar well diffusion method. Significant results were obtained and data were compared with the standard antibiotics penicillium (100 μg/disc) and gentamicin (10 μg/disc). Muhammad et al. [82] measured the antifungal activity of extracts (EtOH and Aq.) of *M. pudica* leaves against *T. verrucosum*, *M. ferrugineum*, *T. shoenleinii*, *T. rubrum*, *M. canis*, *T. concentricum*, *T. soudanense,* and *M. gyseum* at four different concentrations (150, 200, 250, and 300 mg). *T. verrucosum*, *M. ferrugineum*, *T. shoenleinii*, *M. canis*, *T. soudanense,* and *M. gyseum* were sensitive to EtOH extract.

Thakur et al. [142] determined the antimicrobial activity of hydroalcoholic extract of *M. pudica* leaves against *E. coli*, *S. aureus*, *P. aeruginosa,* and *B. cereus* using the disc diffusion method. The extract showed significant results at 25, 50, and 100 mL/disk concentrations. Le Thoa et al. [143] measured the antibacterial activity of Aq. and EtOH extracts of *M. pudica* leaves and stems using the agar well diffusion method. The EtOH extract showed significant zones of inhibition against different strains (*E. coli =* 11 mm, *S. aureus =* 19 mm, *B. cereus =* 17 mm, *S. typhi* = 16 mm), while the Aq. extract significantly inhibited *S. aureus* = 14 mm and *B. subtilis* = 15 mm. The results were compared with standard chloramphenicol. Dhanya and Thangavel [144] measured the antimicrobial potential of MeOH extracts of *M. pudica* leaves, flowers, and roots against *S.aureus*, *E. coli*, and *Pseudomonas* sp. using the disc diffusion method. Zones of inhibition shown by the extract of the leaves in decreasing order were: *S. aureus* (23.5 mm) > *E. coli* (20 mm) > *Pseudomonas* sp*s* (14 mm). The flower extract showed activity against *Pseudomonas* sp. 22.5 > *E. coli* 14 > *S. aureus* 12 mm. The root extract also showed significant activity against *R. solani* (29 mm) *> A. niger* (21 mm) *> M. phaseolina* (17.7 mm). Ahuchaogu et al. [145] screened the antimicrobial potential of the EtOH extract of *M. pudica* whole plant against *S. aureus*, *P. aeroginosa*, *E. coli*, *M. smegmatis,* and *E. faecalis* at various concentrations (25, 50, and 100 mg/disc). At 100 mg/disc, maximum antimicrobial activity was observed and a comparison was made with standard chloramphenicol. Chukwu et al. [146] determined the antimicrobial activity of absolute EtOH extract of *M. pudica* whole plant against the tested microorganisms (*A. flavus* and *T. rubrum*) at three different concentrations (25, 50, and 100 mg/mL). At 100 mg/mL, the extract was very found to be highly active against *A. flavus* and *T. rubrum* (100 mg/mL = 22 and 17 mm, respectively). Rosado-Vallado et al. [23] screened the antimicrobial potential of MeOH and Aq. extracts of *M. Pigra* leaves against various microorganisms (*S. aureus*, *E. coli*, *P. aeruginosa*, *B. subtilis*, *A. niger,* and *C. albicans*) using the agar–well diffusion method. Itraconazole (0.025 mg/µL), nystatin (50 IU/mL), and amikacin (0.03 mg/mL) were used as positive controls for bacteria, yeast, and fungi. Both plant extracts were found to be active against *P. aeruginosa*, *C. albicans*, *S. aureus*, and *B. subtilis* and inactive against *E. coli* and *A. niger.* De Morais et al. [147] determined the antifungal activity of 60% MeOH, DCM, and EtOAc fractions of *M. pigra* leaves by measuring the minimum inhibitory concentration (MIC) values against dermatophyte strains (*T. mentagrophytes*, *E. floccosum*, *M. gypseum*, and *T. rubrum*). The MeOH extract showed the lowest MIC values against all dermatophytes (1.9 to 1000 mg/mL). DCM, EtOAc, and Hex fractions showed significant results. Jain et al. [60] determined the in vitro antimicrobial activity of EtOH extracts and fractions (Aq., CF, PE, and BZ) of *M. hamata* whole plant against *E. coli, K. pneumonia*, *P. aeruginosa*, *S. aureus*, *P. vulgaris*, *A. flavus*, *F. moniliforme*, and *R. bataticola* by disc diffusion method. At 500 mg/disc, the EtOH extract and Aq. fraction inhibited the growth of all bacteria and fungi, although the activity of the Aq. fraction was less than that of the EtOH extracts. PE was found to be active against fungi. Ali et al. [148] measured the antimicrobial activity of crude Hex and MeOH extracts of *M. hamata* whole plant. The Hex extract showed potent % growth inhibition against *B.* cereus (29.75%), *C. diphteriae* (1.40%), *P. aeroginosa* (74.11%), *A. niger* (30.50%), *M. canis* (36.21%), and *M. phaseolina* (89.95%), while the MeOH extract also showed potent activity against *B.* cereus (59.49%), *C*, *diphteriae* (30.16%), *E. coli* (6.31%), *S. sonii* (73.13%), *P. aeroginosa* (32.74%), *S. typhi* (16.84%), *S. pyogenes* (57.18%), *T. longifuses* (67.26%), *P. boydii (*95.10%), *M. canis* (45.31%), *T. simii* (75.00%), *F. solani* (54.75%), and *T. schoenleinii* (84.18%). Standard ampicillin and rifampicin showed 99–100% growth inhibition. Mahmood et al. [5] investigated the antimicrobial potential of crude MeOH extract of *M. pigra* leaves against *E. coli*, *B. subtilis*, *P. aeruginosa*, *K. pneumonia*, *A. niger,* and *A. flavus* by agar tube diffusion and agar tube dilution methods for bacteria and fungi, respectively. The plant showed significant inhibition of *E. coli*, *P. aeruginosa*, and *K. pneumonia* bacteria and minor activity against *B. subtilis*, while no activity was observed against fungi. Silva and his colleagues reported the antimicrobial potential of the EtOH extracts of *M. verrucosa* and *M. pteridifolia* bark via the minimum inhibitory concentration (MIC) values against *S. aureus*, *E. coli*, *C. albicans*, *T. interdigitale*. *M. verrucose,* and *M. pteridifolia,* showing lower MIC values of 250 μg/mL and 500 μg/mL*,* respectively, against *S. aureus* [137] (Table 2).

### 5.2. Antioxidant Activity

Magalhães et al. [149] determined the antioxidant potential of the EtOH extract and various fractions (*n*-hex, DCM, EtOAc, and HyOH) of *M. tenuiflora* leaves, twigs, barks, and roots using DPPH and ABTS radical scavenging activities. The EtOH extract showed the lowest EC_50_ values against DPPH (EC_50_ = 132.99 μg/mL) and ABTS (EC_50_ = 189.14 μg/mL) radicals. The EtOAc fraction proved to have potent antioxidant activity against DPPH (EC_50_ = 141.20 μg/ mL) and ABTS (EC_50_ = 273.00) radicals. Silva and colleagues determined the antioxidant potential of the EtOH extract of *M. tenuiflora* bark using DPPH and ABTS scavenging assays. The plant showed potent scavenging effects against DPPH and ABTS radicals, with IC_50_ values of 17.21 and 3.57 μg/mL, respectively. The results were compared with Trolox [137]. Almalki [30] reported the antioxidant potential of *M. pudica* leaves (Hex extract) by using the DPPH, hydroxyl, nitric oxide, and superoxide radical scavenging assays. The extracts showed significant scavenging effects at concentrations between 5 and 25 mM against DPPH (IC_50_ = 20.83 mM), hydroxyl (IC_50_ = 19.37 mM), nitric oxide (IC_50_ = 21.62 mM), and superoxide (IC_50_ = 22.19 mM) radicals, while the standards butylated hydroxytoluene and vitamin C showed excellent antioxidant potential as compared to the plant extract. Lee et al. [150] determined the antioxidant potential of hydrophilic extracts (ACE-Aq.-AA (8.0 mL, 70:29.5:0.5) of *M. pudica* leaves using oxygen radical absorbance capacity (ORAC) and DPPH free radical scavenging assays. The extract showed significant results in the ORAC (1187.9 = μmol TE g^−1^ FW) and DPPH (EC_50_ = 243.2 mg kg^−1^) assays, while the total vitamin C content was found to be 259 μg/g FW. Durgadevi and Karthika [62] measured the antioxidant activity of the Aq. extract of *M. pudica* leaves using the H_2_O_2_ scavenging assay. Different concentrations (0.2, 0.4, 0.6, 0.8, and 1.0%) of the extract showed (34.6, 39.4, 49.6, 54.6, and 58.3%) significant antioxidant activity. The results were compared with standard thiobarbituric acid. Das et al. [151] determined the antioxidant potential of the MeOH extract of *M. pudica* leaves via DPPH free radical scavenging assay. The IC_50_ values of extracts and ascorbic acid were found to be 126.71 and 20.13 μg/mL, respectively, while the total antioxidant capacity of the extract was IC_50 =_ 5.038 mg/g AAE. Chimsook [152] screened the antioxidant activity levels of PE, EtOAc, absolute EtOH, and Aq. extract of *M. pudica* leaves using ABTS assay. The extracts showed significant results (EC_50_; PE = 40.6, EtOAc = 27.2, absolute EtOH = 73.8, Aq. = 13.2 μg/mL), while standard ascorbic acid showed EC_50_ = 11.5 μg/mL. Parmar et al. [31] screened the antioxidant activity of the HyEtOH extract and L-mimosine compound of *M. pudica* whole plant (stems, leaves, roots, and flower buds) using the DPPH free radical scavenging assay. L-mimosine treatment exhibited lower antioxidant activity than the extract.

Jose et al. [153] screened the in vitro antioxidant activity of isolated flavonoids from EtOAc-soluble fractions of *M. pudica* whole plant by using DPPH and hydroxyl radical scavenging assays. Significant DPPH radical scavenging was observed (IC_50_ = 56.32 μg/mL) as compared to the reference standard (ascorbic acid IC_50_ = 21.11 μg/mL). The isolated flavonoid also showed significant % inhibition at concentrations of 20–140 µg/mL, while ascorbic acid showed significant % inhibition. Ittiyavirah and Pullochal [154] measured the antioxidant activity of the EtOH extract of *M. pudica* whole plant by using H_2_O_2_ and superoxide scavenging assays. The plant showed significant H_2_O_2_ scavenging (IC_50_ = 19 mg/mL), while standard ascorbic acid showed IC_50_ = 5.2 mg/mL. Significant superoxide scavenging was also observed (IC_50_ = 80.4 mg/mL) and gallic acid was used as the standard (IC_50_ = 50.10 mg/mL). Tunna et al. [46] determined the antioxidant potential of the MeOH extract and fractions (*n*-hex, EtOAc, ACE, and MeOH) of *M. pudica* aerial parts using the DPPH free radical scavenging assay. The plant showed significant DPPH radical scavenging and the results were compared with standard ascorbic acid (IC_50_ = 20.13 μg/mL). Silva et al. [32] measured the total phenol and antioxidant potential of the EtOH extract and EtOAc fraction of *M. caesalpiniifolia* leaves using the DPPH free radical scavenging assay. The results for the total phenol and antioxidant activity showed a concentration of 46.8 g gallic acid eq./kg with an antioxidant activity of 35.3 g vitamin C eq./kg in the EtOH extract and 71.50 g gallic acid eq./kg with an antioxidant activity of 65.3 g vitamin C eq./kg in the EtOAc fraction. Rakotomalala et al. [155] determined the antioxidant capacity of the HyMeOH extract of *M. pigra* leaves using DPPH free radical scavenging activity and oxygen radical absorbance capacity (ORAC) assays. The extract showed significant antioxidant potential (DPPH = 1268 and ORAC = 2287 µmol TE/g) as compared to the standard drugs chlorogenic acid (DPPH = 2927 and ORAC = 11.939 µmol TE/µmol) and quercetin (DPPH = 6724 µmol TE/µmol and ORAC = 22,218 µmol TE/µmol).

Saxena et al. [29] determined the in vitro antioxidant properties of the EtOH extract and sub-fractions (EtOAc and diethyl-ether) of *M. hamata* whole plant using DPPH free radical and H_2_O_2_ scavenging assays. The EtOH extract (76.01%) and EtOAc and diethyl-ether sub-fractions (96.63%) showed % inhibition of DPPH scavenging at 100 μg/mL concentration, while the standard drug ascorbic acid showed 93.52% inhibition. The EtOH extract (67.81%) and EtOAc and diethyl-ether sub-fractions (88.43%) showed significant H_2_O_2_ scavenging activity at 100 μg/mL. The results were compared to the standard drug ascorbic acid (86.87%). Chandarana et al. [33] determined the antioxidant potential of cycloHex, EtOAc, and MeOH extracts of *M. hamata* stem using DPPH free radical and ABTS scavenging assays. The MeOH (IC_50_ = 0.70 μg/mL), EtOAc (IC_50_ = 0.85 μg/mL), cycloHex (IC_50_ = 0.95 μg/mL) extracts showed significant DPPH free radical scavenging as compared to standard ascorbic acid (IC_50_ = 0.60 μg/mL). In the ABTS scavenging assay, the MeOH extract showed the highest scavenging activity (IC_50_ = 0.35 μg/mL), followed by EtOAc (IC_50_ = 0.37 μg/mL) and cycloHex extracts (IC_50_ = 0.40 μg/mL), while the standard drug ascorbic acid showed the highest activity (IC_50_ = 0.32 μg/mL). Singh et al. [156] screened the antioxidant activity levels of PE, CF, BuOH, and Aq. extracts of *M. hamata* (stem, leaves, roots, and seeds) using a DPPH free radical scavenging assay. Different extracts of *M. hamata* showed significant DPPH scavenging levels, as represented by IC_50_ values (leaves, 51.30–56.50 μg/mL; stem, 51.80–61.80 μg/mL; roots, 26.33–73.16 μg/mL; seeds, 16.60–51.16 μg/mL). Jiménez et al. [157] determined the antioxidant activity of *M. albida* whole plant with the help of various assays (DPPH radical scavenging, ferric reducing antioxidant power (FRAP), Trolox equivalent antioxidant capacity (TEAC), oxygen radical absorption capacity (ORAC), LDL-C oxidation inhibition). The plant showed significant antioxidant potential in various assays (DPPH = 1540 µmol TE/g, FRAP = 1070 µmol TE/g, TEAC = 1770 µmol TE/g, ORAC = 1870 µmol TE/g). The LDL-C oxidation inhibition assay showed greater than 50% inhibition at 100 µg/mL concentration of the extract. Manosroi et al. [127] investigated the antioxidant efficacy of the Aq. extract of *M. Invisia* leaves using a DPPH free radical scavenging assay. *M. invisa* showed significant free radical scavenging activity (IC_50_ = 0.119 mg/mL), which was 0.49-fold that of the positive control (ascorbic acid). Silva and colleagues determined the antioxidant potential of the EtOH extract of *M. verrucosa* and *M. pteridifolia* bark using DPPH and ABTS scavenging assays. The plant showed potent scavenging effects against DPPH and ABTS radicals, while Trolox was used as the standard antioxidant [137]. (Table 2).

### 5.3. Anticancer Activity

Valencia-Gómez et al. [129] screened the cytotoxicity of biocomposite films made from *M. tenuiflora* cortex and chitosan against (3T3) fibroblasts using MTT assays. Chitosan–*M. tenuiflora* films at different concentrations (100:0, 90:10, 80:20, and 70:30) were used. The cells decreased significantly in the 90:10 and 80:20 chitosan*–M. Tenuiflora* films. Cytotoxicity increased for high–concentration *M. tenuiflora* (70:30) and chitosan films (100:0). Silva and colleagues reported on the cytotoxicity of *M. tenuiflora* bark EtOH extract against four human cancer cell lines (HL-60, HCT-116, PC-3, and SF-295). No activity was observed against any tested cancer lines up to 50 μg/mL concentration [137]. Chimsook [152] reported on the in vitro anticancer activity levels of different extracts (PE, EtOAc, absolute EtOH, and Aq.) of *M. pudica* leaves against three human cancer cell lines derived from lung (CHAGO), liver (HepG_2_), and colon (SW620) samples using an MTT assay. The EtOAc extract was found to be potent (IC_50_ = 29.74 μM) against CHAGO cells, while the EtOAc and absolute EtOH extracts inhibited the SW620 cells, with IC_50_ values of 11.12 and 5.85 μM, respectively. HepG_2_ cell growth was inhibited by EtOAc (IC_50_ = 29.81 μM) and absolute EtOH (IC_50_ = 10.11 μM) extracts. The results were compared with standard amonafide, which showed significant cytotoxicity in CHAGO (IC_50_ = 1.05 μM), SW620 (IC_50_ = 0.32 μM), and HepG_2_ (IC_50_= 1.71 μM) cell lines. Parmar et al. [31] screened the anticancer activity of the Hy-EtOH extracts of *M. pudica* whole-plant samples (stems, leaves, roots, and flower buds) and L-mimosine using MTT assay against the Daudi cell line. At concentrations of 12.5–400 μg/mL, the IC_50_ values were found to be 201.65 μg/mL and 86.61 μM at 72 h for *M. pudica* extract and L-mimosine, respectively. Rakotomalala et al. [155] screened the cell viability and proliferation of smooth muscle in male Wistar rats from HyMeOH extract of *M. pigra* leaves using an MTT assay. No significant effects were observed by the extract (at a concentration of 0.01 to 1 mg/mL) on smooth muscle cell proliferation or cell viability. Saeed et al. [98] measured the antitumor activity of *M. pigra* fruit extract via oral administration. This plant has been used by Sudanese healers against tumors.

Silva et al. [158] screened the anticancer activity of an EtOH extract of *M. caesalpiniifolia* leaves against the human breast cancer cell line MCF-7 by using the SRB assay. The extract at 5.0 μg/mL for 24 h the reduced protein (50%) and cyclophosphamide (30%) contents, while treatment for 48 h reduced protein to 80% and cyclophosphamide to 55%, with the extract showing maximum effect at 320.0 μg/mL, which demonstrates that the extract exhibited cytotoxic effect against MCF-7 cells. Monção et al. [49] reported on the anticancer activity of an EtOH extract and fractions *(n*-Hex, DCM, EtOAc, and Aq.) of *M. caesalpiniifolia* stem bark using an MTT assay against HCT-116, OVCAR-8, and SF-295 cancer cells. The percentage inhibition of cell proliferation for the EtOH extract and *n-*Hex fraction varied from 69.5% to 84.8% and 65.5% to 86.4%, respectively, while the DCM fraction and betulinic acid showed inhibition levels above 86.5% and doxorubicin (at 0.3 μg/mL) >83.0%. EtOAc and Aq. fractions showed minimal inhibition of cell proliferation. Nandipati et al. [122] determined the cytotoxicity of the MeOH extract of *M. rubicaulis* stem against an Ehrlich ascites carcinoma (EAC) tumor model in Swiss albino mice against cancer cell lines (such as EAC, MCF-7, and MDA-MB 435S) using an XTT assay. The extract at a concentration of 200 µg/mL reduced the cytotoxicity of the cell lines (EAC 78.3%, MCF-7 = 79%, MDA-MB 435S = 83%), while standard amoxifen exhibited maximal cytotoxic effects on EAC (99.3%), MCF-7 (95.5%), and MDA-MB 435S (99.4%) cell lines. They also measured the antitumor activity of the *M. rubicaulis (*MeOH extract*)* against an Ehrlich ascites carcinoma (EAC) tumor model in Swiss albino mice who received 100, 200, and 400 mg/kg bw by measuring hematological parameters IRBC, WBC, hemoglobin, and PCV). At a dose of 400 mg/kg, the level of WBC increased while decreases in RBC and PCV were observed as compared to the standard drug 5-FU 20 mg/kg *ip*. Silva and colleagues reported on the cytotoxicity of *M. verrucosa* and *M. pteridifolia* bark EtOH extracts against four human cancer cell lines (HL-60, HCT-116, PC-3, and SF-295). No activity was observed against any of the tested cancer lines up to 50 μg/mL concentration [137] (Table 2).

### 5.4. Antidiabetic Activity

Tunna et al. [46] investigated the antidiabetic potential of MeOH extract and fractions (Hex, EtOAc, ACE, and MeOH) of *M. pudica* aerial parts using α-amylase and α-glucosidase inhibitory assays. The percentages of inhibition in *α*-amylase and *α*-glucosidase inhibitory assays shown by the MeOH extract were found to be 33.86% and 95.65%, while the fractions also showed potent inhibitory effects (Hex = 10.583% and 0.884%, EtOAc = 18.65% and 51.87%, ACE = 15.64% and 16.04%, MeOH = 27.21% and 4.83%). Standard acarbose showed 28.24 and 36.93% inhibition effects, respectively. This study has proven the strong antidiabetic activity of tested extracts, which could lead to future studies with respect to obtaining new antidiabetic agents from *M. pudica*. Piyapong and Ampa [159] screened the hypoglycemic activity of 80% EtOH extract of *M. pudica* whole plant in diabetic male albino Wistar rats using an oral glucose tolerance test (OGTT) and fasting blood glucose test (FBG). In the OGTT, after 30 min of extract administration, the extract (500 mg/kg bw) did not decrease blood glucose (572.83 mg/dL) in diabetic rats as compared to standard glybenclamide (0.5 mg/kg bw = 473.50 mg/dL). In the FBG test, after 1 week of administration, the extract (500 mg/kg bw) decreased the blood glucose level to 421.00 mg/dL, while standard glybenclamide (0.5 mg/kg bw) also significantly decreased blood glucose level (572.67 mg/dL). Konsue et al. [160] determined the antidiabetic activity of Aq. and HyEtOH extracts of *M. pudica* whole plant in diabetic male albino Wistar rats using fasting blood glucose levels (FBG) and hematological values, including red blood cell (RBC), white blood cell (WBC), hemoglobin (Hb), platelet, hematocrit (Hct), mean corpuscular volume (MCV), mean corpuscular hemoglobin (MCH), and mean corpuscular hemoglobin concentration (MCHC) counts, as well as differential white blood cell, lymphocyte, monocyte, neutrophil, and eosinophil counts, at three different concentration (125, 250, and 500 mg/kg bw). At 250 mg/kg bw concentration, Aq. (517.00 mg/dL) and HyEtOH (484.00 mg/dL) extracts significantly decreased fasting blood glucose levels. The results were compared with standard glibenclamide. No effect was observed on RBC, Hb, Hct, platelet, MCH, MCHC, lymphocytes, monocytes neutrophils, or eosinophils, while in diabetic rats the WBC and MCV were decreased by the extract. From this study, it was concluded that use of *M. pudica* Aq. extract could be a potential method of diabetes prevention. Lee et al. [150] determined the enzymatic activity of hydrophilic extracts (ACE–Aq.-AA (8.0 mL, 70:29.5:0.5)) of *M. pudica* leaves using α-amylase and α-glucosidase inhibitory assays. *M. pudica* showed significant inhibition of α-amylase (189.3 μmol AE/g) and α-glucosidase (6.6 μmol AE/g). Acarbose was used as the positive control and statistically significant results were obtained.

Manosroi et al. [127] determined the hypoglycemic activity of *M*. *invisa* leaves (Aq. extract) in normoglycemic and diabetic male ICR mice. Alloxan monohydrate at 75 mg/kg *bw* was injected into the mouse tail vein. After the 3rd day, diabetes was confirmed and various doses (100, 200, and 400 mg/kg bw) of the plant extract were orally given to the 18-h-fasted normal and diabetic mice. Insulin and glibenclamide were used as standards and hypoglycemic effect was measured by decreased fasting blood glucose (FBG). *M. invisa* significantly reduced the fasting blood glucose (FBG) by 14.84% in normoglycemic mice at 1 h with the 200 mg/kg bw dose, which was 0.24- and 0.47-fold the values for insulin and glibenclamide, respectively. *M. invisa* also showed significant FBG reductions of 16.60% and 9.28% at doses of 100 and 400 mg/kg bw at 240 min, which were 0.27- and 0.52-fold the values for insulin and 0.15- and 0.29-fold the values for glibenclamide, respectively. In diabetic mice, *M. invisa* only showed a significant reduction in fasting blood glucose (FBG) of 25.01%, at 180 min with the lower dose of 100 mg/kg bw, which was 0.35-fold that of insulin and 0.55-fold that of glibenclamide, respectively. Ahmed et al. [97] determined the glucose tolerance properties of the MeOH extract of *M. pigra* stem in Swiss albino male mice using the glucose oxidase method. Mice orally received different concentrations of extract (50, 100, 200 and 400 mg/kg/bw) and standard drug glibenclamide (10 mg/kg/bw). After 1 h, all mice orally received 2 g glucose/kg bw. All doses of the extract decreased the concentration of glucose almost 37.84, 39.83, 42.39, and 50.50%, respectively, while glibenclamide reduced the concentration of glucose almost 56.33%. Ao et al. [161] screened the antihyperglycemic activity of the EtOH extract of *M. pigra* roots in albino rats by checking fasting blood glucose (FBG) levels. Diabetes was induced through intraperitoneal injection (160 mg/kg) of alloxan monohydrate. Diabetic albino rats orally received EtOH extract (250 and 500 mg/kg) and glibenclamide (10 mg/kg). In an acute study, administration of the extract at 250 mg/kg concentration showed a significant blood glucose reduction (360.00 mg/dL), while at the 500 mg/kg dose no significant results (391.80 mg/dL) were obtained as compared to diabetic untreated mice; however, the extract showed a significant hypoglycemic effect, while the glibenclamide showed no significant reduction in blood glucose. During prolonged treatment, a fluctuation was observed in the blood glucose levels of the diabetic treated albino rats. The extract (250 and 500 mg/kg) showed a reduction in blood glucose levels (Table 2).

### 5.5. Wound Healing

Choi et al. [162] measured the wound-healing effects of a herbal mixture of *M. tenuiflora* leaves (20%) and *A. vulgaris* (20%) on human keratinocyte (HaCaT), umbilical vein endothelial cells (HUVECs), and mouse fibroblast (3T3-L1) using a scratch test. Fusidic acid was used as the standard. According to the histological study, synthesis of collagen, re-epithelialization, and re-generation of appendages of skin and hair follicles were promoted by the herbal mixture. Immunohistochemical studies showed that blood vessel stabilization, improvement of angiogenesis, and accelerated granulation tissue formation were also achieved through use of the herbal mixture. The herbal mixture can also promote the migration of keratinocytes, endothelial cells, and fibroblasts and the proliferation of macrophages and lymphatic vessels; therefore, the herbal mixture can be used therapeutically for the treatment of cutaneous wounds. Zippel et al. [108] screened the wound-healing efficiency of Aq. extracts and EtOH-precipitated compounds from *M. tenuiflora* bark by measuring the mitochondrial (MTT, WST-1), proliferation (BrdU incorporation), and necrosis (LDH) activities on human primary dermal fibroblasts and HaCaT keratinocytes. The Aq. extract (10 and 100 µg/mL) caused loss of cell viability and proliferation in dermal fibroblasts, while the EtOH-precipitated compound EPC (10 µg/mL) significantly stimulated mitochondrial activity and proliferation of dermal fibroblasts and showed minor stimulation on human kerationocytes at 100 µg/mL. Molina et al. [50] measured the wound-healing activity of 10% powder of *M. tenuiflora* bark in adult humans for external use. The results were found to be significant for inflammation and venous leg ulceration diseases. Arunakumar et al. [163] reported on the wound-healing activity of the MeOH extract of *M. tenuiflora* whole plant by using a chorioallantoic membrane (CAM) model in 9-day-old fertilized chick eggs. The extract increased the numbers of capillaries on the treated CAM surfaces, which might be beneficial for wound healing. Rivera-Arce et al. [24] determined the therapeutic effectiveness of the *M. tenuiflora* cortex extract in the treatment of venous leg ulceration disease. Patients received a hydrogel containing 5% crude extract standardized in a tannin concentration (1.8%). A randomized, double-blind, placebo-controlled clinical trial was conducted. Therapeutic effectiveness was achieved in all patients in the extract group after the 8th treatment week, with ulcer size being reduced by 92% as compared to the control group (Table 2).

### 5.6. Hypolipidemic Activity

Piyapong and Ampa [159] screened the hypolipidemic effects of 80% EtOH extract of *M. pudica* whole plant in diabetic male albino Wistar rats using biochemical data, including total cholesterol (TC), triglyceride (TG), high-density lipoprotein (HDL), and low-density lipoprotein (LDL) levels. In diabetic rats, the levels of TC, TG, and LDL were significantly reduced by plant extract doses and glibenclamide, while plant extract at the dose of 500 mg/kg bw significantly increased HDL. These results indicate that *M. pudica* possesses a hypolipidemic effect in diabetic rats and may lead to decreased risk of cardiovascular disease and related complications. Purkayastha et al. [58] reported on the hypolipidemic effect of an EtOH extract of *M. pudica* leaves in Wistar albino rats with hepatic injury induced by CCl_4_ by measuring biochemical parameters such as triglyceride (TG), total cholesterol (TC), very low density lipoprotein (VLDL), low density lipoprotein (LDL), and high density lipoprotein (HDL) levels. The extract at the dose of 400 mg/kg showed significant decreases in biochemical parameters (TG = 96.8 mg/dL, TC = 98.7 mg/dL, VLDL = 26.9 mg/dL, LDL = 37.4 mg/dL, HDL = 34.3 mg/dL) (Table 2).

### 5.7. Anti-Inflammatory and Hepatoprotective Activity 

Da Silva-Leite et al. [164] determined the healing efficacy of an alcoholic extract prepared from polysaccharides extracted from *M. tenuiflora* barks (EP-Mt) using MeOH/NaOH and EtOH precipitation. The activity was determined in Wistar rat models of acute inflammation (paw edema and peritonitis). The activity was measured with three different doses (0.01, 0.1, and 1.0 mg kg^−1^) of plant extract, with the maximum effect with the 1 mg kg^−1^ concentration as compared to saline. Durgadevi and Karthika [62] reported on the anti-inflammatory activity of an Aq. extract of *M. pudica* leaves by using bovine serum albumin and egg methods. Extracts at different concentrations (0.2, 0.6, 0.6, 0.8, and 1.0%) showed significant anti-inflammatory activity (serum albumin: 51.5, 59.7, 55.7, 71.5, and 83.7%, respectively; egg: 42.5, 39.6, 48.2, 56.7, 65.3, and 76.7%, respectively) when compared with diclofenac sodium. Onyije et al. [165] measured the anti-inflammatory activity of Aq. extract of *M. pudica* leaves on adult male Sprague–Dawley rats with cadmium (CdCl_2_)-induced inflammation of the testes. A sperm analysis was carried out, measuring motility, morphology, and sperm count. Significant activation of sperm motility was observed at different doses of the extract (250 mg/kg = 13.00%; 500 mg/kg = 9.00%) compared with the control group (Aq. = 15.00%). Both doses of the extract showed significant effects on sperm morphology. The sperm counts at different extracts doses (250 mg/kg = 4.18 × 10^6^/cc; 500 mg/kg = 2.54 × 10^6^/cc) were enhanced as compared to control group (12.78 × 10^6^/cc). This study confirmed that *M. pudica* has ethnomedical uses as a therapeutic intervention for infertility; however, when this plant is used as an aphrodisiac, there is also an added benefit of antioligospermia effects. Kumaresan et al. [57] reported on the hepatoprotective activity of a crude powder of *M. pudica* whole plant on male albino rats. Injection in parallel with CCl_4_ and paraffin were given to rats to induce jaundice. Various hepatic parameters such as acid phosphatase (ACP), total bilirubin, gamma glutamyl transferase (γ-GT), alkaline phosphatase (ALP), and lipid peroxide (LPO) levels in tissue, serum, aspartate transaminase (AST), and alanine transaminase (ALT) samples were checked. All of these parameters played roles in liver impairment. A dose of 100 mg/kg of extract powder significantly reduced the levels of all parameters and protected the hepatic cells.

Silva et al. [166] reported on the protective action of HyOH extract and EtOAc fraction of *M. caesalpiniifolia* leaves in adult male Wistar rats suffering from colitis. The HyOH extract (125 and 250 mg/kg) and EtOAc fraction (25 mg/kg) were able to decrease TNF-α immune expression in rats and were found to be effective at lower doses after inducing colitis. The extract showed lower tissue damage at both doses, while the EtOAc fraction was effective at the highest dose (50 mg/kg) only in terms of decreasing COX-2 immune expression. COX-2 and TNF-α played pivotal roles in chronic colitis caused by TNBS. Rakotomalala et al. [155] measured the in vitro anti-inflammatory ability of HyMeOH extract of *M. pigra* leaves in male Wistar rats to reduce TNFα-induced bound vascular cell adhesion molecule 1 (VCAM-1) expression in endothelial cells. The extract at different concentrations (0.01–1 mg/mL) inhibited the induction of VCAM-1 in response to TNFα, with a maximal inhibitory effect of 90% at 1 mg/mL, while the standard drug pyrrolidine dithiocarbamate (200 mM) showed 98% inhibitory effect. In vivo chronic hypoxic PAH cardiac remodeling was also determined in male Wistar rats. Rats were orally treated with extract (400 mg/kg/day) in a hypobaric chamber for 21 days. The extract reduced hypoxic PAH in rats by decreasing pulmonary arterial pressure by 22.3% and pulmonary artery and cardiac remodeling by 20.0% and 23.9%, respectively (Table 2).

### 5.8. Antinociceptive Activity

Patro et al. [167] determined the antinociceptive effects of *M. pudica* leaves (EtOAc extract) on adult Wistar albino rats using AA-induced writhing, hot plate, and tail flick models at three concentration (100, 200 and 400 mg/kg). In the hot plate and tail flick tests, after 30 min the extract and the standard diclofenac sodium significantly increased the analgesic activity. The extract doses of 100, 200, and 400 mg/kg decreased the writhing by 20.18, 33.42, and 43.46% respectively, while the standard diclofenac sodium showed 52.01% writhing inhibition against AA. Ahmed et al. [97] determined the antinociceptive activity of the MeOH extract of *M. pigra* stem in Swiss albino male mice via AA-induced writhing test. Mice orally received various extract doses of 50, 100, 200, and 400 mg/kg/bw, decreasing writhing by 70.01, 74.96, 77.51, and 85.01%, respectively. Aspirin was used as the standard drug.

Rejón-Orantes et al. [106] screened the antinociceptive effects of an Aq. extract of *M. albida* roots in male ICR mice via AA-induced writhing and hot plate tests. In the AA-induced writhing test, the Aq. plant extract at different concentrations (12.5, 25, and 50 mg/kg) and the reference analgesic drug (dypirone, 100 and 500 mg/kg) were administered 60 min before the AA (0.6%) administration. Counts of the writhing responses (abdominal wall contractions and rotation of pelvis followed by extension of hind limb) were carried out during the test (20 min). *M. albida* extract (50 mg/kg) and dypirone (500 mg/kg) prevented the abdominal writhing. This study model was also helpful in an investigation of the opioid system involvement in the antinociceptive effects of the *M. albida* extract. The extract and fentanyl decreased the writhing, although naloxone was only able to antagonize the effects of fentanyl, leaving the antinociceptive potential of the *M. albida* extract intact. Fentanyl seemed to be more potent than the extract. In the hot plate test, pain reaction (hind paw licking and jumping) was determined as the response latency. Before the test, the response latency was determined, after administration of either *M. albida* extract, fentanyl (0.1 mg/kg), or the vehicle (NaCl). Fentanyl (30 and 60 min) after treatment and extract (12.5, 25, and 50 mg/kg) at 60 min from its injection produced significant increases in pain latency (Table 2).

### 5.9. Antiepileptic Activity

Patro et al. [167] measured the antiepileptic effects of EtOAc extract of *M. pudica* leaves on Swiss albino mice using a maximal electroshock (MES)-induced seizure model, PTZ-induced seizure model, and INH-induced seizure model at different extract doses (100, 200 and 400 mg/kg/day). In the maximum electric shock test, the extract at 100, 200, and 400 mg/kg concentrations and the standard drug diazepam (0.4 mg/kg) caused delayed onset of convulsion by 1.87, 2.69, 3.21, and 3.53 s, respectively; as well as decreased duration of convulsion by 68.09, 53.54, 42.21, and 38.89 s, respectively. In the PTZ-induced convulsion test, the extract at different concentrations of 100, 200, and 400 mg/kg and diazepam (04 mg/kg) caused delayed onset of convulsion (5.38, 6.08, 6.98, and 7.81 min, respectively) and decreased duration of convulsion (14.76, 12.65, 11.13, and 9.39 min, respectively). Regarding the INH-induced convulsions, the extract at different concentrations (100, 200, and 400 mg/kg) showed delayed convulsion latency times of 37.21, 45.49, and 58.62 min, respectively; the results were compared with diazepam (04 mg/kg = 69.14 min delayed convulsion latency). Prathima et al. [35] measured the antiepileptic activity of the EtOH extract of *M. pudica* roots in adult Swiss albino mice. Maximal electroshock (MES) and pentylenetetrazole (PTZ)-induced seizures were performed. In the maximal electroshock-induced seizures (MES), the durations of tonic hind limb flexion (THLF), tonic hind limb extension (THLE), clonus, and stupor were noted. In the MES tests, the percentages of inhibition of convulsions in mice at different doses (1000 mg/kg = 42.41%; 2000 mg/kg = 52.35%) were noted, while standard valproate showed 73.86% inhibition at 200 mg/kg. In the pentylenetetrazole (PTZ)-induced seizures, the clonic convulsion onset times, durations of clonic convulsions, and postictal depression were observed for a period of 30 min. The extract (1000 and 2000 mg/kg) significantly decreased the number and duration of myoclonic jerks and clonic seizures and the duration of postictal depression (Table 2). 

### 5.10. Neuropharmacological Activities

Arunakumar et al. [163] determined the anti-Alzheimer’s potential of the MeOH extract of *M. tenuiflora* whole plant by using acetylcholinesterase inhibitory therapy (AChEIs). The results showed that *M. tenuiflora* is a rich source of compounds with potential anti-Alzheimer’s activity. Ttiyavirah and Pullochal [154] measured the antistress activity of EtOH extract of *M. pudica* plant in albino Wistar rats by performing swimming endurance, radial arm maze, Morris Aq. maze, and retention phase tests. Adaptogenic activity was assessed by using oral doses of 500 mg/kg of extract and 2 mg/kg diazepam as the standard compound in the swimming endurance test. In the other three tests, the extract was found to effectively reduced stress as compared to standard D-galactose + piracetam. Significant improvements in memory were observed from the test paradigms for the Morris Aq. Maze and radial arm maze tests. The results from the study indicated that the EtOH extract of *Mimosa pudica* possessed significant antistress activity, along with a potential protective effect against a chronic Alzheimer’s model. Mv et al. [168] measured the neuroprotective effects of *M. pudica* plant in a Parkinson’s male C57BL/6J model at 100 and 300 mg/kg concentrations of extracts using a vertical grid test, horizontal grid test, and immunohistochemistry measurements. In the vertical grid test, the extract at different concentrations (100 and 300 mg/kg) significantly increased the time taken to climb the grid. In the horizontal grid test, the extract decreased the hang time. The extract at 100 and 300 mg/kg doses decreased SYN- and increased DAT- and TH-positive cells. Patro et al. [167] measured the motor coordination activity of the EtOAc extract of *M. pudica* leaves in Swiss albino mice by performing locomotor activity, rotarod, and traction tests with three different extract concentrations (100, 200, and 400 mg/kg), while diazepam at 0.4 mg/kg concentration was used as the standard drug. Significant decreases in locomotor activity were observed. In the rotarod test, the fall time was significantly decreased, while in the traction test, the holding time was also significantly decreased. Kishore et al. [169] screened the CNS activities of Aq. extract of *M. Pudica* leaves in adult albino mice using locomotor activity, elevated plus maze, and rotarod tests at a dose of 200 mg/kg. Percentage changes in locomotor activity were caused by the extract (200 mg/kg = 56.33%) and standard diazepam (0.5 mg/kg = 79.61%). In the elevated plus maze test, the extract (200 kg/mg) and diazepam (0.5 mg/kg) increased the numbers of open arm entries by 67.92% and 78.59% while decreasing the times spent in closed arm positions by 7.32% and 8.64%, respectively. In the rotarod test, the fall times were decreased significantly by the extract (200 kg/mg = 152.1) and diazepam (0.5 mg/kg = 157.6). Mahadevan et al. [170] measured the in vitro neuroprotective effects of the Aq. extract of *M. pudica* whole plant against Parkinson’s disease in SH-SY5Y human neuroblastoma cell lines using cell viability assay or MTT assay. The extract significantly upregulated TH and DAT and downregulated α-synuclein expression in intoxicated cell lines. This disease occurs due to decreases in the dopaminergic neurons and tyrosine hydroxylase (TH) and increases in α-synuclein protein levels.

Rejón-Orantes et al. [106] determined the exploratory and motor coordination activities of the Aq. extract of *M. albida* roots in male ICR mice. Various concentrations of the extract (50, 100, and 200 mg/kg) and the vehicle NaCl were given to mice before tests. For exploratory activity, an open field test was performed to evaluate the locomotor activity of mice. Locomotory activity (number of lines crossed by the animal) was recorded for 5 min. The extract produced a significant decrease in the number of lines crossed by the animal as compared to the vehicle. For motor coordination activity, the rotarod test was performed. The number of falls from the rolling rod were recorded during the test (3 min). The number of falls from the rotarod were significantly increased after supplementation of Aq. extract from the roots of *M. albida* (100 and 200 mg/kg) as compared to the vehicle. Rejón-Orantes et al. [106] reported on the anxiolytic activity of Aq. root extract of *M. albida* in male ICR mice by using elevated plus maze and hole board tests. In the elevated plus maze test, the extract at various concentrations (3.2, 12.5, 25, and 50 mg/kg), dypirone (1 mg/kg), and the vehicle (NaCl) were administrated to mice before testing. In the beginning, the mice were placed on the central plate facing the open arms. Then, the time spent (%) on the open arms was calculated. Significant increases in exploration of open arms in the elevated plus maze test were caused by diazepam. Regarding the hole board apparatus, animals were placed in position and head dippings were counted. Head dippings were significantly enhanced by the diazepam. At all extract doses, *M. albida* showed no significant effect in either test (Table 2).

### 5.11. Antiallergic and Antihyperurisemic Activity

Lauriola and Corazza [113] determined the antiallergic activity of the glyceric acid extract of *M. tenuiflora* bark in a non-atopic 30-year-old woman who had developed acute eczema of the neck in the retroauricular and laterocervical areas. The plant extract was applied on her skin and patch tests were performed. *M. tenuiflora* potentially soothed the skin with its good antimicrobial properties. Sumiwi et al. [171] measured the antihyperurisemic activity of the EtOH (70%) extract of *M.* *pudica* leaves in vitro and ex vivo in Swiss Webster mice (*Mus musculus*). The IC_50_ values for the inhibition of uric acid formation with *M. pudica* tablet, *M. pudica* extract, and allopurinol were 68.04 ppm, 32.75 ppm, and 18.73 ppm, respectively. The ex vivo results showed that *M. pudica* tablet at 125 mg/kg of body weight and the extract reduced uric acid levels in hyperurisemic mice by 36% and 43%, respectively. Mimosa pudica tablets at 125 mg/kg of bodyweight inhibited uric acid formation in hyperuricemic mice; therefore, this pharmaceutical dosage form could be proposed as an antihyperurisemic drug (Table 2).

### 5.12. Larvicidal, Antiparasitic, and Molluscicidal Activity

Oliveira et al. [172] measured the effects of *M. tenuiflora* leaves and stem on the larval establishment of *H. contortus* in sheep. The rate of larval establishment was not reduced by the leaves, but stem intake caused a 27.9% reduction; however, no significant reduction was observed. Bautista et al. [173] determined the antiparasitic activity of Hex, MeOH, and ACE extracts of *M. tenuiflora* leaves against *E. histolytica* and *G. lamblia.* The extracts showed significant inhibition (Hex: IC_50_ = 65.9 and 80.2 μg/mL; ACE: IC_50_ = 80.7 and 116.8 μg/mL; MeOH: 73.5 and 95.5 μg/mL) against both *E. histolytica* and *G. lamblia*, but against *G. lamblia.* Santos et al. [174] determined the molluscicidal activity of the EtOH extract of *M. tenuiflora* stems against the snail species *Biomphalaria glabrata.* The extract showed excellent activity at the 100 µg/mL concentration (LC_90_ = 62.05 mg/L; LC_50_= 20.22 mg/L; LC_10_= 6.59 mg/L). Shamsuddini et al. [175] determined the effects of *M. tenuiflora* stem extracts against human leishmaniases by using an MTT assay and by counting parasites with various concentrations (10, 100, 500, and 1000 micg/mL) of *M. tenuiflora* extracts. Different concentrations of *M. tenuiflora* extract have different effects on the multiplication of *Leishmania protozoa* in culture medium. The multiplication of promastigotes was found to be suppressed at 1000 and 500 micg/mL concentrations. This finding suggested that *M. tenuiflora* extract contains both inhibitory and acceleratory effects on *Leishmania* growth in vitro. Surendra et al. [176] determined the larvicidal action of Aq. extract of *M. pudica* leaves against *Aedes aegypti* larvae at different doses (250, 500, 750, 1000, and 2000 μg/m). The potential was determined at 0, 1, 2, 3, 4, 6, 12, and 24 h and the percentage mortality rates were calculated. Percentage mortality was approximately zero at all concentrations over 24 h. The Aq. extract was found to possess poor larvicidal actions; thus, it can be concluded that *Mimosa pudica* was not suitable for larvicidal actions. Brito et al. [177] reported in vivo anthelmintic (AH) activity of *M. caesalpiniifolia* leaf powder supplementation against nematodes (*Haemonchus*, *Trichostrongylus*, and *Oesophagostomum)* in male goats. Goats were given a *M. caesalpiniifolia* leaf powder that was rich in condensed tannins (days 1–7 and 14–21). After 28 days, the worm burden was estimated. Post-mortem worm counts indicated a decreased in *Haemonchus* adult worm burden (57.7%) in goats. For the CT group, no anthelmintic effect against *Oesophagostomum* was observed; thus, to control gastrointestinal nematode (GIN) infections in goats, feeding with dry *M. caesalpiniifolia* leaves proved encouraging (Table 2).

### 5.13. Antispasmolytic, Antivenom, and Antiviral Activity

Lozoya et al. [135] screened the antispasmolytic activity of BuOH, EtOAc, and MeOH extracts of *M. Tenuiflora* in guinea pig and mouse models. BuOH, EtOAc, and MeOH at 30.0 μg/mL showed significant results by increasing the muscular tonus and frequency of contraction of the uterus. Increases in muscular tonus in the stomach in rats and relaxation of the ileum in guinea pigs were observed. Bitencourt et al. [40] measured the neutralizing capacity of the extract of *M. tenuiflora* bark on the inflammation induced by *Tityus serrulatus* scorpion venom in male BALB/c mice. Animals were inoculated intravenously with saline, Aq. extracts (20, 30, or 40 mg/kg) and fractions, DCM, butyl alcohol, and EtOAc (40 mg/kg). The EtOAc fraction showed potent inhibition against inflammatory cells. The EtOAc fraction showed 83, 67, and 86% inhibition at doses of 20, 30, and 40 mg/kg, respectively. The Aq. extract showed 76% cell inhibition at a dose of 30 mg/kg. Jain et al. [60] determined the in vivo antiviral activity of EtOH extract and fractions (Aq., CF, PE, and BZ) of *M. hamata* whole plant against *H. Simplex*, *poliomyelitis,* and *V. stomatitis* using the plaque inhibition method. The EtOH extract was found to be active against all three viruses. CF and PE were found to be active against *V. stomatitis*. None of the fractions were active against poliomyelitis (Table 2).

## 6. Toxicological Studies of the Genus Mimosa Concerning Hemolysis, Antimutagenic, Genotoxic, and Teratogenic Effects

Magalhães et al. [149] determined the toxicity of the EtOH extract and fractions (Hex, DCM, EtOAc, and HyOH) of *M. tenuiflora* leaves, roots, twigs, and barks using non-specific toxicity to *A. salina* L. and cytotoxicity to African green monkey kidney (Vero) cells via MTT assay. Only the HyOH fraction killed 50% of the nauplii (LC_50_ = 793.70 μg/mL). The fraction of *M. tenuiflora* with the highest antioxidant potential (FATEM) was not toxic to *A. salina* L. (LC_50_ ˃ 1000.00 μg/mL) or Vero cells (CC_50_ = 512.6 μg/mL). Meckes-Lozoya et al. [135] investigated the hemolytic effects of BuOH, EtOAc, and MeOH extracts of *M. tenuiflora* stem bark against enterocytes. BuOH and EtOAc extracts at 250.0 g/mL and MeOH at 500.0 μg/mL increased hemolysis by 74%, 48%, and 68%, respectively. Furthermore, de Morais-Leite et al. [136] measured the hemolytic effects of EtOH extract of *M. tenuiflora* bark on human erythrocytes (types A, B, and O). At the concentration of 1000 μg only, hemolysis was observed in erythrocytes type A at 3.0%, but at the concentration of 2000 μg all three human erythrocytes (A, B, and O) presented hemolysis (23.1, 5.17, and 1.08% respectively). Overall, the extract showed low toxicity for the human erythrocyte cells. Silva and colleagues reported the hemolytic potential of EtOH bark extract of *M. tenuiflora* against human RBCs at various concentrations (250, 500, and 1000 μg/mL). At the 1000 μg/mL concentration, highest hemolysis was observed (90%). While at low concentrations (125, 62.5, 31.25, 15.8 μg/mL), no activity was observed [137] (Table 3).

Prathima et al. [35] measured the acute toxicity of the EtOH extract of *M. pudica* roots in adult Swiss albino mice at different doses (0.5, 1, 2, 4, and 5 g/kg p.o.). There was no mortality amongst the mice treated with the graded dose of extract up to a dose of 5000 mg/kg at a duration of 72 h. Cadmium (Cd) is a well-recognized pollutant with great neuroendocrine-disrupting efficacy. It damages the hypothalamic–pituitary–testicular axis in mature male Wistar rats. The aqueous (Aq.) extract of *M. pudica* leaves was administered orally to rats at a dose of 200 mg/kg for 40 consecutive days. At the end of the analysis period, the extract was used as a therapeutic intervention for infertility [178]. The acute toxicity of EtOAc [167] and EtOH [58] extracts of *M. pudica* leaves on adult Wistar albino rats was determined. No mortality or signs of toxicity were observed at the dose of 2000 mg/kg. Nghonjuyi et al. [22] measured the in vivo toxicity of HyOH extracts of *M. pudica* leaves in Kabir chicks. Single doses of HyOH extracts were administered orally at doses ranging from 40 to 5120 mg/kg for the acute toxicity test. No death was recorded at doses lower than 2560 mg/kg. Very low hypoactivity was observed at the extract dose of 5120 mg/kg. In the sub-chronic study, these extracts were given orally as a single administration to chicks at doses of 80, 160, 320, and 640 mg/kg/day for 42 days. No toxicity was observed with oral sub-chronic low dose administration. Das et al. [151] measured the cytotoxicity of the MeOH extract of *M. pudica* leaves using a brine shrimp lethality bioassay. The LC_50_ of the extract was found to be 282.4 μg/mL, whereas the reference standard vincristine sulphate exhibited an LC_50_ of 0.45 μg/mL. The results of the above findings clearly demonstrated that the leaves of *M. pudica* showed mild cytotoxic properties. Olusayo et al. [179] screened the acute toxicity of the EtOH extract of *M. pigra* roots in adult Wistar rats. No mortality was observed at 5000 mg/kg, which showed that the plant is relatively safe. In sub-acute toxicity tests, three groups of adult Wistar rats were given different concentration of the extract of 250, 500, and 1000 mg/kg/bw, which corresponded to 1/20th, 1/10th, and 1/5th of the 5000 mg/kg dose, respectively. To determine the biochemical (ast, alt, alp, total protein, cholesterol, urea, creatinine, and total bilirubin) and hematological (pcv, rbc, and hb) parameters, rat blood samples were collected on the 29th day. The results of the study showed that there were significant increases in packed hemoglobin, cell volume, and red blood cell count at different extract doses (500 and 1000 mg/kg). The extract produced no significant changes in the levels of total bilirubin, total cholesterol, total protein, aspartate aminotransferase, or alkaline phosphatase in any of the groups that were treated, although a significant increase in the level of alanine transaminase was observed. Serum levels of urea and creatinine were not affected. The findings of this study showed that the roots of *M. pigra* may be safe at doses below 500 mg/kg but may pose toxicological risks at doses greater than 500 mg/kg, with the liver being most affected with prolonged usage. Monção et al. [180] reported on an in vivo toxicological and androgenic evaluation of the EtOH extract of *M. caesalpiniifolia* leaves in adult male Wistar rats using body weight loss and serum biochemical parameters (ALP, AST, urea, and creatinine). In the toxicological evaluation, the extract induced a body weight loss at the highest tested dose (750 mg/kg). No androgenic activity was observed at any dose level (250, 500, or 750 mg/kg). Monção et al. [180] reported on the in vitro cytotoxicity of the EtOH extract of *M. caesalpiniifolia* leaves using an MTT assay in murine macrophages and a brine shrimp lethality assay in *Artemia salina*. The extract showed LC_50_ values of 1765 µg/mL against *Artemia salina* and 706.5 µg/mL against murine macrophages. Rejón-Orantes et al. [106] reported acute toxicity of Aq. extract of *M. albida* roots in male ICR mice. Different doses (3.2, 12.5, 25.50, 100, 200, 300, and 400 mg/kg) of *M. albida* extract were given to various mice groups and their mortality rates were recorded until 48 h; no mortality was observed. Nandipati et al. [122] reported acute toxicity of MeOH extract (500–4000 mg/kg) of *M.* *rubicaulis* stem against Swiss albino mice, administered by oral gavage. No mortality was witnessed at the dose of 4000 mg/kg. Silva and colleagues reported the hemolytic potential of the EtOH bark extracts of *M. verrucosa* and *M. pteridifolia* against human RBCs at various concentrations (250, 500, and 1000 μg/mL). For the *M. verrucosa* extract at the 1000 μg/mL concentration, the highest hemolysis rate was observed (100%). At low concentrations (125, 62.5, 31.25, and 15.8 μg/mL), no activity was observed [138], while M. *pteridifolia* showed no hemolysis with any tested concentrations (Table 3).

Silva et al. [181] measured the mutagenic and antimutagenic effects of crude EtOH extract of *M. tenuiflora* stem bark against *S. typhimurium* strains (TA97, TA98, TA100, TA102) using the Ames test. No mutation was induced in any of the strains at concentrations of 50 and 100 μg/mL of extract. The extract showed antimutagenic effects in all strains, although no antimutagenic effect was observed in TA98. The genotoxicity of crude EtOH extract of *M. tenuiflora* stem bark using a micronucleus test in the peripheral blood of albino Swiss mice has been reported [181]. The extract (100 to 200 mg/kg) and cyclophosphamide (reference drug, 50 mg/kg) were given to mice, whereby the extract at 100 and 200 mg/kg increased the numbers of micronucleus by 8.75 and 9.91, respectively, as compared to cyclophosphamide (50 mg/kg = 43.5). Medeiros et al. [182] determined the teratogenic effects of *M. tenuiflora* seeds in pregnant Wistar rats, whereby a 10% dose of *M. tenuiflora* seeds was given to rats in a Brazilian semiarid climate. The extract was given from the 6th to the 21st day of pregnancy. No differences were observed in weight gain in the lungs, heart, liver, or kidneys of rats or in food or Aq. consumption between treated and controlled rats. Ninety bone malformations were observed in 40 of the 101 fetuses, including skeletal malformations such as scoliosis, bifid sternum, cleft palate, and hypoplasia of the nasal bone. Scientists measured [183,184] the teratogenic effects of *M. tenuiflora* in pregnant goats and lambs in the semiarid rangelands of Northeastern Brazil. The four goats fed on fresh green *M. tenuiflora* during pregnancy delivered 4 kids, 3 of which had abnormalities, including cleft lip, ocular bilateral dermoids, unilateral corneal opacity, buphthalmos (with a cloudy brownish appearance of the anterior chamber due to an iridal cyst), and segmental stenosis of the colon. Dantas et al. [185] measured the teratogenic effects of green fresh *M. tenuiflora* in pregnant goats in the semiarid rangelands of Northeastern Brazil. A high frequency of embryonic deaths was observed in pregnant goats if *M. tenuiflora* was ingested in the first 60 days of gestation. Gardner et al. [186] determined the teratogenicity of *M. tenuiflora* leaves and seeds on pregnant rats in Northeastern Brazil. Compounds extracted from *M. tenuiflora* showed higher incidence rates of soft tissue cleft palate and skeletal malformations. Silva et al. [32] measured the antigenotoxic activities of the EtOH extract and EtOAc fraction of *M. caesalpiniifolia* leaves using a comet challenge assay and micronucleus test. The extract at a concentration of 125 mg/ kg bw inhibited oxidative DNA damage in liver cells, which was induced by hydrogen peroxide (H_2_O_2_) in animals intoxicated with cadmium (Cd). Furthermore, the EtOAc fraction decreased the genomic damage and mutagenesis induced by cadmium exposure. The genus *Mimosa* is able to modulate the toxic effects caused by cadmium exposure as a result of antigenotoxic and antioxidant activities in blood and liver cells of rats (Table 3).

**Table 3:** Toxicological studies of the genus *Mimosa* regarding hemolysis, antimutagenic, genotoxic, and teratogenic effects.

Activities	Plant	Plant Part	Extract/Fraction	Assay	Model	Results/Outcome/Response	References
Toxicological studies	*M. tenuiflora*	Leaves, twigs, barks, roots	EtOH extract and fractions (Hex, DCM, EtOAc and HyOH	(1) Non-specific toxicity (2) Cytotoxicity to Vero cells by MTT assay	(1) *Artemia salina* L. (2) Vero cells of African green monkey kidney	Only the HyOH fraction killed 50% of the nauplii (LC50 = 793.70 μg/mL). Fractions of EtOH extract were not toxic to *A. salina* L. (LC50 ˃ 1000.00 μg/mL). Fractions of EtOH extract were not toxic to Vero cells (CC50 = 512.6 μg/mL)	[149]
		Stem bark	BuOH, EtOAc MeOH	In vitro	Erythrocytes	Buthanol 250 μg/mL = 74%; EtOAc 250 g/mL = 48%; MeOH 500 μg/mL = 68%	[135]
			EtOH	Hemolytic assay	Human erythrocytes type A, B, and O	At 1000 μg concentration, only hemolysis of erythrocyte A (3%) was observed, while at 2000 μg concentration, extract showed hemolysis on type A = 23.1%; type B = 5.17%; type O = 1.08%	[136]
		Bark	EtOH	Hemolytic assay	Human RBCs	% hemolysis at 1000 μg/mL = 90%, 500 μg/mL = 35%, 250 μg/mL = 17%	[137]
	*M. pudica*	Roots	EtOH	Acute toxicity	Swiss albino mice	No mortality was observed at extract dose up to 5000 mg/kg	[35]
		Leaves	Aq.	Histoarchitecture parameters	Mature male Wistar rats/cadmium-induced toxicity	Extract doses of 200 mg/kg were found effective	[178]
			EtOAc	Acute toxicity	Adult Wistar rats	No mortality or signs of toxicity were observed at the dose of 2000 mg/kg	[167]
			EtOH	Acute toxicity	Wistar albino rats	No mortality as observed up to the dose level of 2000 mg/kg bw	[58]
			HyOH		In vivo toxicity/Kabir chicks	Very low toxicity observed at high dose of 5120 mg/kg	[22]
						Sub-chronic toxicity observed at doses of 80, 160, 320, and 640 mg/kg	
			MeOH	Brine shrimp lethality bioassay		Extract 1–500 μg/mL; LC50 = 282.3495 μg/mL), standard vincristine sulphate; LC50 = 0.45 μg/mL	[151]
	*M. pigra*	Roots	EtOH	Acute toxicity	Adult Wistar rats	No mortality observed	[179]
				Hematological and biochemical parameters	Adult Wistar rats	Doses greater than 500 mg/kg posed toxicological risks	
	*M. caesalpi-niifolia*	Leaves	EtOH	Body weight and serum biochemical parameters (ALP), AST, urea, and creatinine	In vivo: Male adult Wistar rats	Toxicological evaluation induced a body weight loss, which was observed at the highest tested dose of 750 mg/kg Extract did not show androgenic activity at any doses (250, 500, 750 mg/kg)	[180]
				Brine shrimp and MTT assay	In vitro: *Artemia* *salina* and murine macrophages	LC50 = 1765 mg.L^−1^ (Artemia salina) LC50 = 706.5 mg.L^−1^ (murine macrophages)	[180]
	*M. albida*	Roots	Aq.	Acute toxicity	Male ICR mice	No mortality observed at different extract doses (3.2, 12.5, 25.50, 100, 200, 300, and 400 mg/kg)	[106]
	*M. rubicau-* *lis*	Stems	MeOH	Acute toxicity	Swiss albino mice	Doses (range of 500–4000 mg/kg) did not lead to acute toxicity	[122]
	*M. verruco-* *sa*	Bark	EtOH	Hemolytic assay	Human RBCs	% hemolysis at 1000 μg/mL = 100%, 500 μg/mL = 72%, 250 μg/mL = 43%	[137]
	*M. pteridi-* *folia*	Bark	EtOH	Hemolytic assay	Human RBCs	No activity observed	[137]
Antimutag-enic, genotoxic, and teratogeniceffects	*M. tenuiflora*	Stems, bark	crude EtOH extract	Ames mutagenic/antimutagenic test	*S. typhimurium* TA 97, TA 98, TA 100, TA 102 strains	Extracts at 50 and 100 μg.mL^−1^ did not induce mutations in any strain Extracts at 50 and 100 μg/mL showed antimutagenic effects in all strains (TA97, TA100, and TA102)	[181]
				Micronucleus test	In vivo: Erythrocytes of albino Swiss mice	Extracts at 100 and 200 μg/mL did not show significant results	
		Seeds	10% of seeds	In vivo	Pregnant Wistar rats (Rattus novergicus)	90 bone malformations were observed in 40 of the 101 rats, including scoliosis, lordosis, and a shorter head	[182]
		Green forage	In vivo	Fed green forage of *M. tenuiflora* throughout gestation period	Pregnant goats and lambs	3 of 4 kids had abnormalities including cleft lip, unilateral corneal opacity, ocular bilateral dermoids, buphthalmos with a cloudy brownish appearance in the anterior chamber due to an iridal cyst, and segmental stenosis of the colon	[184]
		Green forage		In vitro	Pregnant goats	Embryonic deaths were observed	[185]
		Leaves and seeds	Compounds extracted from *M. tenuiflora*		In vitro: Pregnant rats	Soft tissue cleft palate and skeletal malformations were observed in pups	[186]
	*M. caesalpiniifolia*	Leaves	EtOH and EtOAc fraction	Comet, challenge assay, and micronucleus Test	In vivo: Wister rats	Plant extract significantly prevented genotoxicity in liver and peripheral blood cells	[32]

## 7. Conclusions

Herbal medicines are used to cure different ailments worldwide. Drugs are becoming resistant to treatment, so there is a dire need to find novel natural sources of traditional compounds. In this review, we presented ethnogeographical distribution and the traditional, nutritional and pharmacological values of *Mimosa* species. All species showed versatile potential pharmacological activities, such as antimicrobial, antioxidant, anticancer, antidiabetic, wound-healing, hypolipidemic, anti-inflammatory, hepatoprotective, antinociceptive, antiepileptic, neuropharmacological, toxicological, antiallergic, antihyperurisemic, larvicidal, antiparasitic, molluscicidal, antimutagenic, genotoxic, teratogenic, antispasmolytic, antiviral, and antivenom effects. In the future, the plants from this genus should be promising in the development of new drugs. This genus consists of 400 species but only 20–25 are well known, while the rest have not yet been explored; therefore, the species of the genus *Mimosa* may hold potential for drug discovery. The best possible efforts have been made to review and summarize the available information. This review could be a useful tool in assisting researchers in discovering new medicinal benefits of the genus *Mimosa*.

## Figures and Tables

**Table 1:** Ethnotraditional uses of different species of the genus *Mimosa*.

Plant	Plant Parts	Country	Common Names in Different Languages	Uses	References
*M. tenuiflora*	Stem bark	Mexico	Tepescohuite	Used for to treat cutaneous wounds, burns, and inflammations	[9,24,108,109,110,111]
	Stem bark, leaves, flowers	Northeastern Brazil, Venezuela	Skin tree, jurema-preta, calumbi	Used for injury, odontalgia, inflammations, fever, menstrual colic, headache, hypertension, bronchitis, cough, external ulcers	[41,55,76,77,78,79,80,81]
	Root bark	Northeastern Brazil	Jurema preta, black jurema, Vinho de jurema	Used for Jurema, a psychoactive beverage consumed for medicoreligious purposes	[74,75]
	Bark	NortheasternBrazil		Some indigenous tribes use it as a miraculous drink	[55,112]
		Italy		Used for eczema	[113]
	Whole plant	Mexico		Used for hallucinogenic compounds	[114]
*M. pudica*	Whole plant	Cameroon, Mexico	English—“Touch me not”; Urdu—Chhimui; Punjabi—Lajan; Hindi—Lajauni, Chhuimui; Marathi—Lajalu; Gujrati—Lajavanti, Risamani, Lajamani; Bengali—Lajjavanti, Lajaka; Telugu—Mudugudamara; Tamil- Tottavadi, Tottalchurungi; Oriya—Lajakuri; Kannada—Lajjavati, Muttidasenui, Machikegida; Sanskrit—Namaskari, Samanga, Varakranta; Malayalam—Thottavati	Used for to treat headaches, insomnia, depression, anxiety, premenstrual syndrome, hemorrhoids, skin wounds, menorrhagia, diarrhea, rheumatoid arthritis	[50,82,83,84,85]
	Roots	India		Used for to treat fever, dysentery, piles, jaundice, uterine and vaginal illnesses, burning sensation, leucoderma, asthma, inflammations, leprosy, fatigue, blood infections	[86]
	Stem		Touch-me-not	Aphrodisiac properties, antivenom activities, antihepatotoxic effect, diuretic effect, hyperglycemic effect, wound-healing effect	[115]
	Leaves	Bangladesh		Used for to treat piles, diarrhea, persistent dysentery, convulsion of children	[90]
		Philippines	Iloko—Bain bain; Tagalog—Makahiya		[116,117]
	Leaves and roots	India	Namaskari, Lajjalu	In Unani and Ayurvedic methods of medication, *M. Pudica* is used for treatment of ulcers, bile, leprosy, fever, small pox, jaundice, piles, ulcers, inflammation, burning sensations, asthma, hemorrhoids, spasmodic fistula, strangury, hydrocele, scrofula, conjunctivitis, wounds, hemorrhages	[87,88,89,118]
	Whole plant			Used internally to treat vesicle calculi and externally to treat myalgia, rheumatism, uterus tumors, odema-type disorders	[89]
	Roots	Bangladesh		Antivenom effects	[91]
	Bark	China		Treatment of traumatic injury to dissipate blood stasis	[93]
	Herb	China		In women is used in vagina-narrowing solution	[92]
	Paste	China		Used in dental powder to treat gingiva and bad breath	[93]
	Seeds+5 g sugar	India	Chunimui	Venereal diseases	[72]
	Leaves			Used to cure skin infections	
*M. pigra*	Shrub	Africa		Used for asthma, respiratory diseases, diarrhea, typhoid fever, genitourinary tract infections	[94]
	Roots, leaves, stem	Madagascar, tropical Africa, South America, Indonesia		Used for head colds, mouthwash for toothaches, eye medicines	[96]
	Leafy stem fruits	Africa		Antivenom effects	[95]
	Leaves	Mexico		Used in Mayan medicine for treatment of diarrhea	[23]
		Bangladesh		Used to lower blood sugar in diabetic patients and for the treatment of pain	[97]
	Roasted and ground leaves	Indonesia	English name—bashful plant; vernacular name—Alfas	Used to treat a weak heart or weak pulse	[98,99]
*M. caesalipiniifolia*	Bark and flowers	Northeastern Brazil	Cascudo, sabia	Used for bronchitis, skin infections, injuries, inflammation, hypertension, cough, gastritis	[27,77,100,105,119,120,121]
*M. hamata*	Whole plant	India	Jinjani, hooked	Used to treat jaundice, diarrhea, coagulant, fever, dysentery, wounds. Used as a blood-purifier and as a tonic for urinary complaints and piles	[3]
	Leaves			Used for treatment of burns, glandular swelling, sores, piles. Used as a sinus dressing and contraceptive	[101,102]
	Seeds			Blood purifier	[33,103]
*M. rubicaulis*	Whole plant	India	Commonly known as *Mimosa himalayan;* korinda, putta korinda in Telugu	Used to treat leucoderma, leprosy, chronic diarrhea, rheumatism, treatment of snake bite, fungal infections, cuts and wounds	[122]
*M.* *somnians*	Whole plant	Central America	Dormidera, sensitive plant		[104]
*M. bimucronata*	Leaves	Brazil		Cardiovascular, renal system, diuresis, and saluresis treatment in rats	[123,124]
*M. linguis*	Whole plant			Diuretic	[26]
*M. humilis*	Whole plant			Rheumatism treatment	
*M. invisa*	Leaves	Nigeria	Idon zakara, Nila grass	Used to treat bronchitis, and asthma and to relieve tooth pain, has antidiabetic properties	[125,126,127,128]
*M. arenosa*	Bark	Brazil		Used for asthma	[77]
*M. ophthalmo-centra*	Bark	Brazil	Jurema	Used for bronchitis, cough	
*M. verrucosa*	Stem bark	Brazil	Jurema-preta	Used for gastritis, ulcer, asthma, inflammation of the uterus	[77,105]
*M. albida*	Roots, leaves and flowers	Mexico		Used for pain and anxiety, chronic pain	[106]
	Roots	Honduras		Used for abortion	[107]

**Table 2:** Pharmacological studies of different species of the genus *Mimosa*.

Activities	Plant	Plant Part	Extract/ Fraction	Assay	Model	Results/Outcome/Response	References
Antimicro-bialactivity	*M. tenuiflora*	Bark and chitosan	Biocomposite film	Turbidimetry method	*E. coli*, *M. lysodeikticus*	Chitosan/*M. Tenuiflora* film showed potent antibacterial activity	[129]
		Wood	Pyroligneous acid	Agar diffusion method	*E. coli*, *C. albicans,* *P. aeruginosa*, *S. aureus*, *C. neoformans*	Significant inhibition obtained at all concentrations	[130]
		Bark	HyOH extracts	Agar diffusion method	*E. coli*, *S. aureus*, *E. aerogenes*, *K. pneumoniae*, *Providencia*, *P. aeruginosa*, *P. Mirabilis*, *S. sonnei*, *S. pyogenes*, *Staphylococcus* spp.	Actively inhibited the growth of bacteria	[131]
			EtOH	Agar dilution method, time-kill assay	*S. aureus*	Bactericidal effect was observed	[132]
			EtOH 95%	Disc diffusion method	*E. coli*, *B. subtilis*, *M. luteus*, *P. oxalicum*	Significant results obtained. Active doses of extract: *E. coli* = 5.0 ug/disk; *B. subtilis* = 10.0 ug/disk; *M. luteus* = 20.0 ug/disk; *P. oxalicum* = 10.0 ug/disk	[133]
			EtOH 95%	Minimum inhibitory concentration (MIC)	*S. epidermidis*, *A. calcoaceticus*, *S. aureus*, *M. luteus*, *E. coli*, *K. pneumonia*, *P. aeruginosa*, *C. albicans*	Active doses of extract *S. epidermidis*, *A. calcoaceticus* = MIC >10.0 μg/mL, *S. aureus*, *M. luteus* MIC = 10.0 μg/mL, *E. coli*, *K. pneumonia* MIC = 20.0 μg/mL, *C. albicans* MIC = 70.0 μg/mL	[134]
			BuOH	Well diffusion method	*S. aureus*, *E. coli*	Active doses of extract: *S. aureus* = 5.0 mg/well; *E. coli* = 15.0 mg/well	[135]
			MeOH		*S. aureus*, *E. coli*	Active doses of extract: *S. aureus* = 5.0 ug/well; *E. coli* = 30.0 ug/well	
			EtOAc		*E. coli*, *C. albicans*	Active doses of extract: *E. coli* = 10.0 mg/well; *C. albicans* = 30.0 mg//well	
			EtOH extract	Minimal inhibitory concentration (MIC) and minimal bactericidal concentration (MBC)	*S. aureus* ATCC 25.925 and ATCC 25.213, *E. coli* ATCC 8859 and ATCC 2536, *P. aeruginosa* ATCC 25.619	For *S. aureus* (ATCC 25.925) and *P. aeruginosa* (ATCC 25.619), MIC = 128 and MBC = 256 μg/mL), respectively; for *S. aureus* ATCC 25.213 (MIC = 512, MBC = 1024 μg/mL), *E. coli* ATCC 8859, and *E. coli* ATCC 2536 (MIC = 1024, MBC ≥1024 μg/mL)	[136]
			EtOH	Minimum inhibitory concentration (MIC)	*S. aureus*, *E. coli*, *C. albicans*, *T. interdigitale*	Active dose of extract showed MIC against *S. aureus* = 15.6 μg/mL,*E. coli* = 1000 μg/mL, *C. albicans* = 156.28 μg/mL, *T. interdigitale* = 156.28 μg/mL	[137]
	*M. pudica*	Leaves	EtOH extract	Disc diffusion method	*S. aureus*, *B. subtilis*, *C. albicans*	ZOI; *S.aureus* = 4.61%, *B. subtilis* = 4.5%, *C. albicans* = 1.96%	[116,117]
			Aq.	Disc diffusion method	*E. coli*, *staphylococcus* sp., *Bacillus* sp., *Pseudomonas* sp. *Streptococci* sp.	ZOI: *E. coli =*18 mm *> Bacillus* sp*. =* 12.5 mm > *Pseudomonas* sp. = 12 mm *>Staphylococcus* sp. = 11 mm > *Streptococci* sp*. =* 9 mm	[138]
			ACE, EtOAc, PE, Aq.	Well diffusion method	*E. coli*, *P. aeurogiosa*, *L. bacillus*, *S. typhi*, *S. aureus*, *P. foedians*, *F. oxysporum*, *P. variotii*	Concentration (30–120 μL/mL) antibacterial activity of extract increased with increasing dose of extract. At 120 μL/mL, more ZOI was observed	[139]
			Aq.	Agar well diffusion method	*B. cereus*, *E. coli*, *P. valgaris*, *S. aureus*, *P. auroginosa*, *A. flavus*, *A. niger*, *Fusarium* sp., *Penicillium* sp., *A. terreus*,	ZOI at 25 and 100 mg concentration against *B. cereus =* 5 and 10 mm, *E. coli =* 9 and 22 mm, *P. valgaris =* 0 and 9 mm, *P. auroginosa =* 4 and 15 mm, *S. aureus* = 11 and 18 mm; *A. flavus* = 5 and 25 mm, *A. niger* = 5 and 14 mm, *A. terreus* = 8 and 17 mm, *Fusarium* sp. = 6 and 15 mm, *Penicillium* sp. = 6 and 11 mm	[62]
			MeOH	Disc diffusion method	*P. aeruginosa*, *S. aureus*, *V. harveyi*	At 100 μL concentration ZOI showed by extract against *P. aeruginosa* = 8.66 mm, *S.aureus* = 10.66 mm, *V. harveyi* = 8.00 mm; ampicillin used as standard	[140]
			MeOH	Agar well diffusion method	*B. subtilis*, *S. aureus*, *P. vulgeris*, *S. typhi*, *P. aeroginosa*	ZOI *B. subtilis* = 14 mm; *S. aureus* = 12 mm, *P. vulgeris* = 11 mm; *S. typhi* = 15 mm; *P. aeroginosa* = 12 mm; penicillium (100 μg/disc) and gentamicin (10 μg/disc) used as standards	[141]
			EtOH and Aq.	Agar well diffusion method	*T. verrucosum*, *M. ferrugineum*, *T. schoenleinii*, *T. rubrum*, *M. canis*, *M. gypseum, T. concentricum*, *T. soudanense*	ZOI at 150–300 mg/mL in EtOH extract = 0–6 mm, in Aq. extract = 0–7 mm	[82]
			MeOH	Disc diffusion and agar well diffusion method	*M. tuberculosis*	ZOI in disc diffusion method = 7.00 mm, agar well diffusion method = 4.33 mm, streptomycin = 25 mm	[140]
			HyOH	Disc diffusion method	*E. coli*, *S. aureus*, *P. aeruginosa, B. cereus*	Extract showed significant results at 25, 50, and 100 mL/disk concentrations.	[142]
		Leaves and stems	EtOH and Aq. extracts	Agar well diffusion method	In vitro/*E.coli*, *S.aureus*, *B. cereus*, *S. typhi*	At 100 μL of EtOH extract, ZOI against *E. coli =* 11 mm; *S.aureus =* 19 mm; *B. cereus =* 17 mm; *S. typhi* = 16 mm. In Aq. extract, *S. aureus* = 14 mm; *B. cereus* = 15 mm	[143]
		Leaves, flowers, roots	EtOH, CF, MeOH	Disc diffusion method	*S. aureus*, *E. coli*, *Pseudomonas* sp*s*, *M. phaseolina*, *A. niger*, *R. solani*	ZOI in leaves: *S.aureus* = 23.5 mm, *E.coli* = 20 mm, *Pseudomonas* sp*s.* = 14 mm; In flower; *Pseudomonas* sp*s* = 22.5 mm *E.coli* = 14 mm, *S.aureus =* 12 mm; in roots: *R. solani =* 29 mm, *A. niger =* 2 1 mm, *M. phaseolina =* 17.7 mm	[144]
		Whole plant	EtOH 98%	Disc Diffusion method	*S. aureus*, *E. faecalis*, *P. aeroginosa*, *E. coli*, *M. smegmatis*	ZOI at 25 mg/mL concentration; *S. aureus =* 3.5 mg/mL; *P. aeroginosa =* 12.0 mg/mL; *E. coli =* 5.5 mg/mL. At 100 mg/mL concentration *S. aureus =* 9.8 mg/mL; *P. aeroginosa =* 18.0 mg/mL; *E. coli =* 14.0 mg/mL. Standard chloramphenicol at 100 mg/mL showed significant results	[145]
			Absolute EtOH	Disc diffusion method	*A. flavus*, *T. rubrum*	ZOI at 100 mg/mL concentration of extract against *A. flavus =* 22 mm; *T. rubrum =* 17 mm. At 25 mg/mL concentration of extract: *A. flavus =* 13 mm*; T. rubrum* 11 mm	[146]
	*M pigra*	Leaves	MeOH and Aq. extract	Agar well diffusion method	*S. aureus*, *E. coli*, *A. niger*, *P. aeruginosa*, *B. subtilis*, *C. albicans*	Plant was found to be active against all strains except *E. coli*, *A. niger*	[23]
			MeOH 60%	Broth microdilution method	In vitro*: T. mentagrophytes*, *E. floccosum*, *M. gypseum*, *T. rubrum*	All strains showed antifungal activity except *E. floccosum*	[147]
			MeOH 60%, Hex, DCM, EtOAc fractions	Minimal inhibitory concentration (MIC)		Significant results were observed against strains	
			Crude MeOH extract	Agar tube diffusion method	*B. subtilis*, *A. niger*, *P. aeruginosa*, *E. coli*, *K. pneumonia*, *A. flavus*.	Potent activity was obtained against bacteria, while no activity was found against fungi	[5]
	*M. hamata*	Whole plant and callus tissue	EtOH extract and its fractions (Aq, CF, PE, BZ)	Disc diffusion method	*E. coli*, *K. pneumoniae*, *A. flavus*, P. aeruginosa, *P. vulgaris*, *S. aureus*, *F.moniliforme*, *R. bataticola*	EtOH extract and Aq. fraction showed significant activity against all tested strains PE found to be active against fungi	[60]
		Whole plant	Crude Hex, MeOH extracts		*B.* cereus, *C. diphteriae*, *E. coli*, *A. niger*, *T. simii*, *P. aeroginosa*, *M. canis M. phaseolina*, *P. boydii*, *M. canis*, *F. solani*, *T. schoenleinii*, *S. sonii*, *S. typhi*, *S. pyogenes*, *T. longifuses*, *P. mirabillis*, *S. boydii*, *S. aureus*, *S. pyogenes*, *R. solani*, *C. albicans*	% inhibition of crude Hex extract against *B.* cereus (29.75%), *C. diphteriae* (1.40%), *P. aeroginosa* (74.11%), *A. niger* (30.50%), *M. canis* (36.21%), and *M. phaseolina* (89.95%). Crude MeOH extract; *B.* cereus, (59.49%), *C. diphteriae* (30.16%), *E. coli* (6.31%), *S. sonii* (73.13%), *P. aeroginosa* (32.74%), *S. typhi* (16.84%), *S. pyogenes* (57.18%), *T. longifuses* (67.26%), *P. boydii* (95.10%), *M. canis* (45.31%), *T. simii* (75.00%), *F. solani* (54.75%), *T. schoenleinii* (84.18%), while standard ampicillin and rifampicin showed 99–100% growth inhibition	[148]
	*M.* *verrucosa*	Barks	EtOH	Minimum inhibitory concentration (MIC)	*S. aureus*, *E. coli*, *C. albicans*, *T. interdigitale*	Active dose of extract showed MIC against *S. aureus =* 250 μg/mL, *E. coli =* 1000 μg/mL, *C. albicans =* 1250 μg/mL, *T. interdigitale =* 78.13 μg/mL	[137]
	*M.* *pteridi-* *folia*	Barks	EtOH	Minimum inhibitory concentration (MIC)	*S. aureus*, *E. coli*, *C. albicans*, *T. interdigitale*	Active dose of extract showed MIC against *S. aureus* = 500 μg/mL, *E. coli* = 1000 μg/mL, *C. albicans* = 625 μg/mL, *T. interdigitale* = 312.5 μg/mL	[137]
Antioxidant activity	*M.* *tenuiflora*	Leaves, twigs, barks, roots	EtOH extract and fractions (Hex, DCM, EtOAc, HyOH	DPPH radical and ABTS radical cation scavenging assay		In EtOH extract: DPPH (EC_50_) = 132.99 μg/mL; ABTS (EC_50_) = 189.14 μg/mL. EtOAc fraction: DPPH (EC_50_) =141.20 μg/mL; ABTS (EC_50_) = 273.00 μg/mL	[149]
		Bark	EtOH	DPPH radical and ABTS radical cation scavenging assay		DPPH (IC50) = 17.21 μg/mL, ABTS (IC_50_) = 3.75 μg/mL	[137]
	*M. pudica*	Leaves	*n-*Hex	DPPH, OH, NO, and superoxide radical scavenging assays		*n-*Hex at 5–25 mM concentration showed DPPH (IC_50_ = 20.83 mM); OH (IC_50_ = 19.37 mM); NO (IC_50_ = 21.62 mM), O_2_^-^ (IC_50_ = 22.19 mM); BHT and vitamin C used as standards	[30]
			ACE-Aq-AA (8.0 mL, 70:29.5:0.5)	ORAC assay, DPPH free radical scavenging activity		ORAC = 1187.9 μmol TE g^−1^ FW), DPPH EC_50_ = 243.2 mg/kg), vitamin C content = 259.1 μg/g FW)	[150]
			Aq. 1.0%	Hydrogen peroxide scavenging, reducing power assays		H_2_O_2_ scavenging for 0.2 and 1.0% extract concentration = 34.6 and 58.3%, respectively. Reducing power for 0.2 and 1.0% extract = 59.8 and 94.7% activity, respectively; while standard thiobarbitaric acid extract at 0.2 and 1.0% = 59.7 and 86.3% activity, respectively	[62]
			MeOH	DPPH free radical scavenging assay		DPPH scavenging; IC_50_ = 126.71 μg/mL, ascorbic acid; IC_50_ = 20.13 μg/mL; total antioxidant capacity of extract = 5.038 mg (mg AAE/g)	[151]
			PE, EtOAc, EtOH, Aq. extract	ABTS assay		PE; EC_50_ = 40.6 μg/mL, EtOAc; EC_50_ = 27.2 μg/mL; EtOH; EC_50_ = 73.8 μg/mL; Aq.; EC_50_ = 13.2 μg/mL, ascorbic acid; EC_50_ = 11.5 μg/mL	[152]
		Whole plant	HyEtOH extract and L-Mimosine compound	DPPH radical scavenging assay		At concentration 31.25–250 μg/mL, HyEtOH extract (IC_50_ = 103.88 μg/mL), L-mimosine (IC_50_ = 233.06 μM)	[31]
			Isolated flavonoids from EtOAc-soluble fractions of MeOH	DPPH free radical, OH radical scavenging assays		DPPH = % inhibition at 20–140 µg/mL, standard ascorbic acid at 0–100 µg/mL, OH radical scavenging at 240–1000 µg/mL; quercetin standard at 0–300 µg/mL showed significant results	[153]
			EtOH	Hydrogen peroxides and superoxide scavenging assay		H_2_O_2_ assay; extract (IC_50_ = 19 mg/mL) ascorbic acid; IC_50_ = 5.2 mg/mL, O_2_^-^ assay; extract (IC_50_ = 80.4 mg/mL), gallic acid; IC_50_ = 50.10 mg/mL	[154]
		Aerial parts	MeOH extract and fractions (Hex, EtOAc, ACE, and MeOH)	DPPH free radical scavenging activity		DPPH assay; MeOH extract = (IC_50_ 7.18 μg/mL) Fractions; MeOH = (IC_50_ 158.4 μg/mL); Hex = (IC_50_ 92.30 μg/mL); EtOAc = (IC_50_ 49.59 μg/mL); ACE = (IC_50_ 45.63 μg/mL). Ascorbic acid = IC_50_ 20.13 μg/mL	[46]
	*M. caesalpi-* *niifolia*	Leaves	EtOH and EtOAc fractions	DPPH free radical scavenging assay		EtOH extract = 35.3 g vitamin C.eq /kg; EtOAc fraction = 65.3 g vitamin C eq/kg	[32]
	*M. pigra*	Leaves	HyMeOH	DPPH free radical scavenging activity, oxygen radical absorbance capacity (ORAC)		DPPH = 1268 µmol TE/g; ORAC = 2287 µmol TE/g; chlorogenic acid (reference drug), DPPH = 2927 µmol TE/µmol; ORAC = 11.939 µmol TE/µmol; quercetin (reference drug); DPPH = 6724 µmol TE/µmol; ORAC = 22,218 µmol TE/µmol	[155]
	*M. hamata*	Whole plant	Crude EtOH extract and sub-fraction (EtOAc and diethyl ether)	DPPH radical scavenging, hydrogen peroxide scavenging assay		% inhibition at 100 μg/mL; DPPH scavenging; crude EtOH extract = 76.01% EtOAc, diethyl ether sub-fraction = 96.63%; ascorbic acid = 93.52%; H_2_O_2_ scavenging; extract = 67.81% EtOAc, diethyl ether sub-fraction = 88.43%; ascorbic acid = 86.87% scavenging activity	[29]
		Stem	MeOH, cycloHex, and EtOAc	DPPH free radical scavenging assay, ABTS scavenging assay	In vitro	DPPH radical scavenging assay IC_50_; MeOH = 0.70 μg/mL, EtOAc = 0.85 μg/mL, cycloHex = 0.95 μg/mL, ascorbic acid = 0.60 μg/mL; ABTS assay IC_50_; MeOH = 0.35 μg/mL, EtOAc = 0.37 μg/mL; cycloHex = 0.40 μg/mL; ascorbic acid = 0.32 μg/mL	[33]
		Leaves, stems, roots, seeds	PE, CF, BuOH, and Aq.	DPPH free radical scavenging assay		IC_50_; leaves = 51.30–56.50 μg/mL, stems = 51.80–61.80 μg/mL, roots = 26.33–73.16 μg/mL, seeds = 16.60–51.16 μg/mL	[156]
	*M. albida*	Whole fresh plant	Aq.	DPPH radical, ferric reducing antioxidant power (FRAP), Trolox-equivalent antioxidant capacity (TEAC), oxygen radical absorption capacity (ORAC), low-density lipoprotein (LDL) assays		DPPH = 1540 µmol TE/g FRAP = 1070 µmol TE/g, TEAC = 1770 µmol TE/g, ORAC = 1870 µmol TE/g LDL = 50% inhibition	[157]
	*M. invisa*	Leaves	Aq. Extract	DPPH radical scavenging assay	In vitro	Aq. extract IC_50_ = 0.119 mg/mL Ascorbic acid IC_50_ = 0.058 mg/mL	[127]
	*M. verrucosa*	Bark	EtOH	DPPH radical and ABTS radical cation scavenging assay	-	DPPH (IC_50_) = 33.22 μg/mL, ABTS (IC_50_) = 4.91 μg/mL	[137]
	*M. pteridifo-* *lia*	Bark	EtOH	DPPH radical and ABTS radical cation scavenging assay		DPPH (IC_50_) = 51.82 μg/mL, ABTS (IC_50_) = 4.88 μg/mL	[137]
Anticancer activity	*M. tenuiflora*	Biofilm of cortex and chitosan	Biocomposite film	(3T3) fibroblast by MTT assays		Cells decreased significantly in the 90:10 and 80:20 chitosan*/M. tenuiflora* films. Cytotoxicity increased in high-concentration *M. tenuiflora* (70:30) and chitosan films (100:0)	[129]
		Bark	EtOH	MTT assay	Human tumor cell lines HL-60 (acute myeloid leukemia), HCT-116 (Colorectal carcinoma), PC-3 (prostate adenocarcinoma), SF-295 (glioblastoma)	Extract displayed IC_50_ ≥ 50 μg/mL against all cell lines, while no activity was observed against HCT-116	[137]
	*M. pudica*	Leaves	PE, EtOAc, EtOH, Aq. extract	MTT assay	In vitro: Human cancer cell lines from lungs (CHAGO), liver (HepG_2_), colon (SW620)	CHAGO cell; absolute EtOAc (IC_50_ = 29.74 μM), SW620 cell; EtOAc (IC_50_ = 11.12 μM) and absolute EtOH (IC_50_ = 5.85 μM); HepG_2_ cell; EtOAc (IC_50_ = 2 29.81 μM) and absolute EtOH (IC_50_ = 10.11 μM)	[152]
		Whole plant	HyEtOH extract and L-mimosine compound	MTT assay	In vitro*:* Daudi cell line	At concentration of 12.5–400 μg/mL; Extract showed IC_50_ = 201.65 μg/mL and L- Mimosine (IC_50_ = 86.61 μM)	[31]
	*M. pigra*	Leaves	HyMeOH	MTT assay	In vitro: Male Wistar rats, endothelial and aortic smooth muscle cell	Extract (0.01–1 mg/mL) showed no significant effect on cellular viability/proliferation	[155]
		Fruit			Intake orally	Active against tumor	[98]
	*M. caesalpi-* *niifolia*	Leaves	EtOH	SRB assay	Human breast cancer cell line MCF-7	Extract showed maximum effect at 320.0 μg/mL.	[158]
		Stems, bark	EtOH extract, *n*-Hex, DCM, EtOAc, Aq. fractions	MTT assay	HCT-116 (colon), OVCAR-8 (ovarian), SF-295 (glioblastoma) tumor cell lines	EtOAc and Aq. fractions showed minimum inhibition of cell proliferation while EtOH showed = 69.5–84.8%, *n*-Hex fraction = 65.5–86.4%, DCM fraction and betulinic acid ≤ 86.5%., doxorubicin ≥ 83.0%	[49]
	*M. rubicauli-* *slam*	Stems	MeOH	Hematological parameters (hemoglobin content, RBC, WBC, PCV)	Ehrlich ascites carcinoma (EAC) tumor model, Swiss albino mice	At a dose of 400 mg/kg, the level of WBC increased, with decreases in RBC, PCV as compared to standard drug 5-FU 20 mg/kg,*iP*	[122]
				XTT assay (EAC, MCF-7, MDA-MB 435S cell lines	In vivo*:* Swiss albino mice	At dose of 200 mg/kg; IC_50_ values of extract; EAC = 72.326 µg/mL, MCF-7 = 69.692 µg/mL, MDA-MB 435S = 80.565 µg/mL; IC_50_ tamoxifen (stranded), EAC = 22.42µg/mL, MCF-7 = 20.7 µg/mL, MDA-MB 435S = 20.87µg/mL	
	*M. verrucosa*	Barks	EtOH	MTT assay	Human tumor cell lines HL-60 (acute myeloid leukemia), HCT-116 (colorectal carcinoma), PC-3 (prostate adenocarcinoma), SF-295 (glioblastoma)	Extract displayed IC_50_ ≥ 50 μg/mL against all cell lines	[137]
	*M. pteridifo-lia*	Barks	EtOH	MTT assay	Human tumor cell lines HL-60 (acute myeloid leukemia), HCT-116 (colorectal carcinoma), PC-3 (prostate adenocarcinoma), SF-295 (glioblastoma)	Extract displayed IC_50_ ≥ 50 μg/mL against all cell lines	[137]
Antidiabetic activity	*M. pudica*	Aerial parts	MeOH extract and fractions (Hex, EtOAc, ACE, and MeOH)	α-Amylase inhibitory assay, α-glucosidase inhibitory assay	In vitro	% inhibition in α- amylase and α-glucosidase inhibitory assays showed by MeOH extract = 33.86 and 95.65% (fractions; Hex = 10.583 & 0.884%, EtOAc = 18.65 and 51.87%, ACE = 15.64 and 16.04%, MeOH= 27.21 and 4.83%), respectively. Standard acarbose = 28.24 and 36.93%	[46]
		Whole plant	80% EtOH	Oral glucose tolerance test (OGTT) and fasting blood glucose test	Streptozotocin (STZ)-induced diabetic male albino Wistar rats	Extract 500 mg/kg bw did not decrease blood glucose in STZ-induced diabetic rats as compared to 0.5 mg/kg bw. After 1 week, blood glucose reduction shown by extract (500 mg/kg bw) = 421.00 mg/dL, glybenclamide (0.5 mg/kg bw) = 572.67 mg/dL	[159]
		Whole plant	Aq. and HyEtOH extracts	Fasting blood glucose test (FBG)	Streptozotocin (STZ)-induced diabetic male albino Wistar rats	Significantly decreased FBG levels At 250 mg/kg bw concentration of Aq. = 517 mg/dL, Hy-EtOH = 484.00 mg/dL. At 500 mg/kg bw concentration Aq. = 309.88 mg/dL, HyEtOH = 484.00 mg/dL, glibenclamide (0.5 mg/kg *bw*) = 419.00 mg/dL	[160]
		Leaves	ACE–Aq.–AA (8.0 mL, 70:29.5:0.5)	α-Amylase and α-glucosidase inhibitory assay	In vitro	α-Amylase = 189.3 μmol AE/g; α-glucosidase = 6.6 μmol AE/g. Acarbose was used as the positive control.	[150]
	*M. pigra*	Aq. extracts	Leaves	Fasting blood glucose (FBG)	Normoglycemic male ICR mice	Significant FBG reduction in aq. extract (200 mg/kg/bw) = 14.84%, (100 mg/kg bw) = 16.60% (400 mg/kg bw) = 9.28%, insulin (0.5 IU /kg) = 54.05%, glibenclamide (1 mg/kg) = 31.39%	[127]
					Diabetic male ICR mice	Extract (100 mg/kg bw) = 25.01%, insulin (0.5 IU /kg) = 56.62%, glibenclamide (1 mg/kg) = 18.51%	
		Stems	MeOH	Glucose oxidase method	Swiss albino male mice	Significant blood glucose reduction by extract at 400 mg/kg/bw = 50.50%, glibenclamide (10 mg/ kg/bw) = 56.33%	[97]
		Roots	EtOH	Fasting blood glucose (FBG)	Albino rats	Significant blood glucose reduction in acute study extract (250 and 500 mg/kg) = 360.00 and 391.80 mg/dL respectively; glibenclamide (10 mL/kg) = 485.8 mg/dL. In prolonged study, extract (250 and 500 mg/kg) = 140.00 and 125.00 mg/dL, respectively. Glibenclamide (10 mL/kg) = 273.60 mg/dL	[161]
Wound-healing effects	*M. tenuiflora*	Leaves	Herbal mix of leaf extract (20%) and *A. Vulgaris* (20%)	In vitro/scratch assay	In vivo: Human keratinocyte (HaCaT) and umbilical vein endothelial cells (HUVECs), mouse fibroblast 3T3-L1 cells	Rapid wound healing observed	[162]
		Bark	Aq. extracts, EtOH-precipitated compounds (EPC)	Mitochondrial activity (MTT, WST-1), proliferation (BrdU incorporation), necrosis (LDH)	In vitro: Human primary dermal fibroblasts and HaCaT keratinocytes	Aq. extract (10 and 100 µg/mL) loss of cell viability was observed proliferation in dermal fibroblasts. EPC (10 µg/mL) only stimulated mitochondrial activity and proliferation of dermal fibroblasts. EPC at 100 µg/mL showed minor stimulation of human kerationocytes	[108]
		Whole plant	10% concentration		Adult human external use	Significant results observed	[50]
		Whole plant	MeOH	Chorioallantoic membrane (CAM) model	Ex vivo: Fertilized chick eggs	Significant results observed	[163]
			Crude EtOH cortex extract standardized in its tannin concentration (1.8%)	Double-blind, randomized, placebo-controlled clinical trial	Patients diagnosed withvenous leg ulceration (VLU)	Ulcer size was reduced by 92%	[24]
Hypolipide-mic activity	*M. pudica*	Whole plant	80% EtOH	TC, TG, HDL, LDL	Streptozotocin (STZ)-induced diabetic male albino Wistar rats	Extract at 500 mg/kg bw increased HDL level = 46.33 mg/dL but decreased TC = 111.67 mg/dL, TG = 121.67 mg/dL, LDL = 41.00 mg/dL in the diabetic rats as compared to standard glybenclamide (0.5 mg/kg *bw*)	[159]
		Leaves	EtOH	TG, TC, VLDL, LDL, HDL	Wistar albino rats, induced hepatic injury by (CCl_4_)	Extract at the dose of 400 mg/kg showed significant decreases in TG 96.8 mg/dL, TC = 98.7 mg/dL, VLDL = 26.9 mg/dL, LDL = 37.4 mg/dL, HDL = 34.3 mg/dL	[58]
Anti-infla-mmatory and hepato-protective activities	*M. tenuflora*	Bark	MeOH/NaOH and EtOH precipitation polysaccride	Edematogenic effect	Wistar rats of acute inflammation (paw edema and peritonitis)	Edematogenic effect at 1 mg/kg^−^concentration of polysaccharides extracted from *M. tenuiflora* bark was = 40x as compared to saline, while inhibited by L-NAME = 52%, dexamethasone = 26%	[164]
	*M. pudica*	Leaves	Aq.	Bovine serum albumin, egg	In vitro	Reduced activity of extract in serum albumin at 0.2 and 1.0% concentration = 59.7 and 83.7%, respectively; while drug diclofenac sodium = 51.5%; In egg at 0.2 and 1.0% conc = 39.6 and 76.7%, respectively. Standard drug diclofenac sodium = 42. 5%	[62]
		Leaves	Aq.	Sperm motility, sperm morphology, sperm count	Adult male Sprague–Dawley rats, cadmium-induced testicular damage	(1) Significant activation of sperm motility shown by extract at 250 mg/kg = 13.00%; 500 mg/kg = 9.00%, control group (Aq.) = 15.00%. (2) Both doses showed significant effects on sperm morphology. (3) Sperm counts were significantly increased at 250 mg/kg = 4.18 × 10^6^/cc; 500 mg/kg = 2.54 × 10^6^/cc; control group = 12.78 × 10^6^/cc	[165]
		Whole plant	Crude powder	ALP, ACP, LPO, γ-GT, AST, ALT.	Male albino rats, induced jaundice by (CCl_4_)	100 mg/kg dose of extract significantly reduced the levels of all parameters and protected the hepatic cells	[57]
	*M. caesalpi-* *niifolia*	Leaves	HyOH extract, EtOAc fraction	Histopathological analysis	In vivo: Adult male Wistar rats	HyOH extract (125 and 250 mg/kg), EtOAc fraction (25 and 50 mg/kg) were effective	[166]
	*M. pigra*	Leaves	HyMeOH	TNFα-induced	In vitro: male Wistar rats, endothelial cells	Extract (0.01–1 mg/mL) inhibited 90% and pyrrolidine dithiocarbamate (200 mM) inhibited 98% TNFα	[155]
				Chronic hypoxic PAH	In vivo: Male Wistar rats	Decreased pulmonary arterial pressure = 22.3%, pulmonary artery = 20.0%, cardiac remodeling = 23.9% was observed	[155]
Antinoci-ceptive activity	*M. pudica*	Leaves	EtOAc	Hot plate test, tail flick test, AA-induced writhing test	Adult Wistar rats	Hot plate test after 30 min significantly increased analgesic activity by extract at 100 mg/kg = 8.03, 200 mg/kg = 8.51; 400 mg/kg = 8.93; standard diclofenac sod. = 9.66. Tail flick test after 30 mins significantly increased analgesic activity at 100 mg/kg extract = 6.78, 200 mg/kg = 8.16; 400 mg/kg = 7.98; standard diclofenac sod. = 8.11. Significant reduced writhing shown by extract at 100, 200, and 400 mg/kg = 20.18, 33.42, and 43.46%, respectively; standard diclofenac sodium = 52.01%	[167]
	*M. Pigra*	Stems	MeOH	AA-induced writhing test	Swiss albino mice male	Significant reduction in writhing; extract (400 mg/kg/bw) = 85.01%, aspirin (400 mg/kg/bw) = 59.97%	[97]
	*M. albida*	Roots	Aq.	AA-induced writhing test, hot plate test	Male ICR mice	Extract (50 mg/kg) and dypirone (500 mg/kg) reduced writhing. Fentanyl (0.1 mg/kg) and extract at variable concentrations showed pain latency	[106]
Antiepile-ptic activity	*M. pudica*	Leaves	EtOAc	Electric shocks, PTZ-induced convulsions, INH-induced convulsions	Swiss albino mice	In electric shock test, extract at 100, 200, and 400 mg/kg and diazepam 04 mg/kg exhibited delayed onset time of convulsion = 1.87, 2.69, 3.21, and 3.53 s, as well as decreased duration of convulsion of 68.09, 53.54, 42.21, and 38.89 s, respectively. In PTZ-induced convulsion test, extract at 100, 200, and 400 mg/kg and diazepam 04 mg/kg showed delayed onset time of convulsion = 5.38, 6.08, 6.98, and 7.81 min and decreased duration of convulsion of 14.76, 12.65,11.13, and 9.39 min, respectively. In INH-induced convulsion test, extract at 100 mg/kg = 37.21, 200 mg/kg = 45.49, 400 mg/kg = 58.62 min, diazepam 04 mg/kg = 69.14 min delayed convulsion latency	[167]
		Roots	EtOH	Maximal-electroshock-induced seizures (MES) and pentylenetetrazole (PTZ)-induced seizures	Adult Swiss albino mice	In MES, the % inhibition of convulsions in mice at different doses (1000 mg/kg = 42.41%; 2000 mg/kg = 52.35%) were noted and standard valproate showed 73.86% inhibition at 200 mg/kg. In PTZ-induced seizure, the clonic convulsion onset time, duration of clonic convulsions, and postictal depression were observed for a period of 30 min. Extract (1000 and 2000 mg/kg) showed significant decreases in numbers and durations of myoclonic jerks, clonic seizures, and postictal depression.	[35]
Neurophar-macological activities	*M. tenuiflora*	Whole plant	MeOH	Acetylcholinesterase inhibitory therapy	Ex vivo	Plant showed anti-Alzheimer’s properties	[163]
	*M. pudica*	Whole plant	EtOH	Swimming endurance test, radial arm maze, Morris Aq. maze and retention phase	In vivo: Albino Wister rats suffering from chronic Alzheimer’s	Oral dose of 500 mg/kg of extract and 2 mg/kg diazepam standard were effective in swimming endurance test. In all other tests, extract was found to effective in reducing stress as compared to standard D-galactose + Piracetam	[154]
		Whole plant	Aq.	Vertical grid test, horizontal grid test, immunohistochemistry	In vitro: Male C57BL/6J mice	In vertical grid test, extract at 100 and 300 mg/kg significantly increased time taken to climb the grid. In horizontal grid test, extract at 100 and 300 mg/kg decreased the hang time, extract at 100 and 300 mg/kg decreased SYN and increased DAT and TH-positive cells	[168]
		Leaves	EtOAc	Locomotor activity, rotarod and traction test	Swiss albino mice	Significant decreased in locomotor activity was observed in extract at 100 mg/kg = 362.43, 200 mg/kg = 331.24, 400 mg/kg = 276.12, diazepam 04 mg/kg = 152.41.In rotarod test, the fall time was decreased significantly by extract at 100 mg/kg = 173.45, 200 mg/kg = 149.13, 400 mg/kg = 121.43, diazepam 04 mg/kg = 19.21. In traction test, the holding time of mice was significantly decreased by extract at 100 mg/kg = 4.83, 200 mg/kg = 3.47, 400 mg/kg = 2.75, diazepam 04 mg/kg = 1.03	[167]
		Leaves	Aq.	Locomotor activity, elevated plus maze test, rotarod test	Adult Swiss albino mice	% change in locomotor activity shown by extract 200 mg/kg = 56.33%, while standard diazepam 0.5 mg/kg = 79.61%. Elevated maze test; extract 200 kg/mg and diazepam 0.5 mg/kg; increased number of open arm entries = 67.92% and 78.59%; decreased time spent in closed arms = 7.32% and 8.64%. In rotarod test, the fall time was decreased significantly by extract 200 kg/mg = 152.1 and diazepam 0.5 mg/kg 157.6	[169]
		Whole plant	Aq.	Cell viability or MTT assay	In vitro: Human neuroblastoma SH-SY5Y cells	At 300 µg, extract showed IC_50_ = 211.05 μg/mL against parkinsonism	[170]
	*M. albida*	Roots	Aq.	Elevated plus maze hole board test, open field test, rotarod test	Male ICR mice	In both elevated plus maze and hold board tests, the extract showed non-significant results at (3.2, 12.5, 25, and 50 mg/kg concentration, while diazepam (1 mg/kg.ip) showed significant results. In open field test, the significant effects at variable concentrations (50, 100, 200 mg/kg) extract of 200 mg/kg and diazepam (1 mg/kg.ip) showed significant results in rotarod test	[106]
Antiallergic and antihyper-uricemic activities	*M. tenuiflora*	Bark	Glyceric extract	Patch test	Women suffering from acute eczema	Positive result was observed	[113]
	*M. pudica*	Leaves	EtOH 70%	Inhibitory activity assay	In vitro: Male rats (*Rattus norvegicus*)	Inhibition % of uric acid formation in *M. pudica* tablet IC_50_ = 68.04 ppm, extract IC_50_ =32.75 ppm, allopurinol (standard) IC_50_ = 18.73 ppm	[171]
					Ex vivo: Swiss–Webster mice *(Mus musculus*)	Inhibitory activity on uric acid formation shown by *M. pudica* tablet 125 mg/kg = 36%, extract = 43%	
Laravicidal, antiparasitic, and molluscici-dal activities	*M. tenuiflora*	Leaves and stem		Parasitological and histological analysis	Female lambs	No significant effect observed	[172]
		Leaves	Hex, ACE, MeOH	Antiprotozoal assay	In vitro*: E. histolytica*, *G. lamblia*	At concentration of 2.5–200 µg/mL; *E. histolytica and G. lamblia* showed activities in Hex (IC_50_) = 65.9 and 80.2 μg/mL, ACE = 80.7 and 116.8 μg/mL, MeOH = 73.5 and 95.5 μg/mL, respectively	[173]
		Stems	EtOH		In vitro: *Biomphalaria glabrata*	At 100 μg/mL; LC_10_ = 6.59 mg/L; LC_50_ = 20.22 mg/L; LC_90_ = 62.05 mg/L	[174]
				MTT assay and counting parasites	In vitro: Human leishmaniases	Extract of Mimosa at concentrations of 500 and 1000 mg/L rapidly reduced parasite proliferation	[175]
	*M. pudica*	Leaves	Aq.	Larvicidal assay	Larvae (Aedes aegypti)	Poor larvicidal action at dose of 2000 mg/kg	[176]
	*M. caesalpiniifolia*	Leaves	Dry plant leaves and condensed tannin	Worm burden	In vivo: Goats	Significantly controlled the gastrointestinal nematodes (*Haemonchus*, *Tricho strongylus, Oesophagostomum*) in goats	[177]
Antispasmo-lytic, antivenom, and antiviral activities	*M. tenuiflora*	Bark	BuOH, EtOAc, MeOH	In vitro	Guinea pigs and rats	Buthanol, EtOAc, and MeOH at 30.0 μg/mL showed significant results.	[135]
			Aq. extract and fractions (DCM, BuOH and EtOAc)	Envenomation	Male BALB/c mice (in vitro)	Inhibition (%) at 30 mg/kg Aq. extract = 76%; at 40 mg/kg; DCM fraction = 73% butyl alcohol, fraction _=_ 81%; EtOAc fraction = 86%	[40]
	*M. hamta*	Whole plant and callus tissue	EtOH extract, Fractions, Aq., CF, PE, BZ	Plaque inhibition method	In vivo: *H. simplex*, *Poliomyelitis type 1*, *V. stomatitis*	EtOH extract showed significant activities against all three viruses, while PE and CF fractions were found to be active against *V. stomatitis*	[60]

## Data Availability

All the data produced here are available and can be produced when required.

## Abbreviations

BuOH	Butanol
Hex	Hexane
ACE	Acetone
MeOH	Methanol
EtOAc	Ethyl acetate
EtOH	Ethanol
DEE	Diethyl ether
AA	Acetic acid
DM	Dry matter
*M. lysodeikticus*	*Micrococcus lysodeikticus*
*E. aerogenes *	*Enterobacter aerogenes*
*P. aeruginosa*	*Pseudomonas aeruginosa*
*P. Mirabilis*	*Proteus mirabilis*
*C. albicans*	*Candida albicans*
*S. epidermidis*	*Staphylococcus epidermidis*
*M. luteus*	*Micrococcus luteus*
*S. typhi*	*Salmonella typhi*
*P. variotii*	*Paecilomyces variotii*
*P. valgaris*	*Proteus vulgaris*
*A. niger*	*Aspergillus niger*
*T. mentagrophytes*	*Trichophyton mentagrophytes*
*M. gypseum*	*Microsporum gypseum*
*B. subtilis*	*Bacillus subtilis*
*F. moniliforme*	*Fusarium verticillioide*
*C. diphtheria*	*Corynebacterium diphtheria*
*M. phaseolina*	*Macrophomina phaseolina*
*F. solani*	*Fusarium solani*
*S. boydii*	*Shigella boydii*
ZOI	zone of inhibition
ABTS	2,2-azinobis-(3-ethylbenzothiazoline-6-sulfonate)
IC_50_	inhibitory concentration
NO	Nitric oxide
RBC	Red blood cell count
WBC	White blood cell count
PCV	Packed-cell volume
ICR mice	Institute of Cancer Research
EPC	EtOH-precipitated compounds
VLU	venous leg ulceration
TNFα	Tumor Necrosis factor alpha
VCAM-1	vascular cell adhesion molecule 1
TNBS	Trinitrobenzenesulfonic acid
HyOH	Hydroalcohol
Aq.	Aqueous
PE	Pet. Ether
CF	Chloroform
Bz	Benzene
DCM	Dichloromethane
HyMeO	Hydromethanol
HyEtOH	Hydroethanol
*E. coli*	*Escherichia coli*
*S. aureus*	*Staphylococcus aureus*
*K. pneumonia*	*Klebsiella pneumonia*
*S. sonnei*	*Shigella sonnei*
*S. pyogenes*	*Streptococcus pyogenes*
*C. neoformans*	*Cryptococcus neoformans*
*A. calcoaceticus*	*Acinetobacter calcoaceticus*
*L. bacillus*	*Lacto bacillus*
*F. oxysporum*	*Fusarium oxysporum*
*B. cereus*	*Bacillus cereus*
*A. flavus*	*Aspergillus flavus*
*A. terreus*	*Aspergillus terreus*
*E. floccosum*	*Epidermophyton floccosum*
*T. rubrum*	*Trichophyton rubrum*
*P. vulgaris*	*Proteus vulgaris*
*R. bataticola*	*Rhizoctonia bataticola*
*M. canis*	*Microsporum canis*
*P. boydii*	*Pseudallescheria boydii*
*T. schoenleinii*	*Trichophyton schoenleinii*
*R. solani*	*Rhizoctonia solani*
DPPH	2,2-diphenyl-1-picrylhydrazyl
EC_50_	effective concentration
OH	Hydroxide
TE	Trolox equivalent
SRB assay	sulforhodamine B
XTT assay	Cell Proliferation Kit II
CCl_4_	Carbon tetrachloride
CAM	Chorioallantoic membrane
L-NAME	*N*-Nitro-l-arginine methyl ester hydrochloride
COX-2	cyclooxygenase-2
PAH	*Hypoxic pulmonary hypertension*
